# NUDT5 regulates purine metabolism and thiopurine sensitivity by interacting with PPAT

**DOI:** 10.1126/science.adx9717

**Published:** 2025-11-06

**Authors:** Zheng Wu, Phong T Nguyen, Varun Sondhi, Run-Wen Yao, Zhifang Lu, Tao Dai, Jui-Chung Chiang, Feng Cai, Imani M Williams, Eliot B Blatt, Zengfu Shang, Ling Cai, Jing Zhang, Mya D Moore, Islam Alshamleh, Xiangyi Li, Tamaratare Ogu, Lauren G Zacharias, Rainah Winston, Joao S Patricio, Xandria Johnson, Wei-Min Chen, Qian Cong, Thomas P Mathews, Yuanyuan Zhang, Limei Zhang, Ralph J DeBerardinis

**Affiliations:** 1 Children’s Medical Center Research Institute, University of Texas Southwestern Medical Center, Dallas, TX, 75390, USA; 2 Division of Pulmonary and Critical Care Medicine, Department of Internal Medicine, UT Southwestern Medical Center, Dallas, TX, 75390, USA; 3 Department of Biophysics, University of Texas Southwestern Medical Center, Dallas, TX, 75390, USA; 4 Department of Radiation Oncology, University of Texas Southwestern Medical Center, Dallas, TX, 75390, USA; 5 Harold C. Simmons Comprehensive Cancer Center, University of Texas Southwestern Medical Center, Dallas, TX, 75390, USA; 6 Quantitative Biomedical Research Center, Peter O’Donnell Jr. School of Public Health, University of Texas Southwestern Medical Center, Dallas, TX, 75390, USA; 7 McDermott Center for Human Growth and Development, University of Texas Southwestern Medical Center, Dallas, TX, 75390, USA; 8 Department of Biochemistry, University of Nebraska-Lincoln, Lincoln, NE, 68588, USA; 9 Howard Hughes Medical Institute, University of Texas Southwestern Medical Center, Dallas, TX, 75390, USA

## Abstract

Cells generate purine nucleotides through de novo purine biosynthesis (DNPB) and purine salvage. Purine salvage represses DNPB to prevent excessive purine nucleotide synthesis through mechanisms that are incompletely understood. We identified Nudix hydrolase 5 (NUDT5) as a DNPB regulator. During purine salvage, NUDT5 suppresses DNPB independently of its catalytic function but through interaction with phosphoribosyl pyrophosphate amidotransferase (PPAT), the rate-limiting enzyme in the DNPB pathway. The NUDT5-PPAT interaction promoted PPAT oligomerization, suppressed PPAT’s enzymatic activity, and facilitated disassembly of the purinosome, a metabolon that functions in DNPB. Disrupting the NUDT5-PPAT interaction overcame DNPB suppression during purine salvage, permitting excessive DNPB and inducing thiopurine resistance. Therefore, NUDT5 governs the balance between DNPB and salvage to maintain appropriate cellular purine nucleotide concentrations.

Cells coordinate de novo purine biosynthesis (DNPB) and salvage to maintain purine nucleotide pools (1, 2). Inhibiting DNPB promotes purine salvage (3), whereas activating purine salvage represses DNPB to prevent excessive synthesis (4). Perturbing purine homeostasis impairs the purine-pyrimidine balance, causing replication stress, DNA damage, and cell cycle arrest (5). Mutations causing hyperactivity of phosphoribosylpyrophosphate synthetase (PRPS1), the enzyme that produces phosphoribosyl pyrophosphate (PRPP) at the initial step of purine synthesis, drive excessive DNPB through failed feedback inhibition, resulting in hyperuricemia and neurological dysfunction (6, 7).

The ability to engage either DNPB or purine salvage is pivotal for adaptation to stress, including energetic stress. In contrast to salvage, DNPB is energetically demanding and requires metabolic input from the pentose phosphate pathway, nonessential amino acid metabolism, the folate cycle, and oxidative mitochondrial metabolism (1). Impairment of mitochondrial respiration drives a shift from DNPB to purine salvage, preventing unnecessary energy expenditure and enabling cell growth (8). However, a complete understanding of the interplay between DNPB and purine salvage is lacking.

DNPB enzymes associate into dynamic cytosolic complexes called purinosomes that facilitate purine synthesis by channeling intermediates from one enzyme to the next (9–11). Purine deprivation induces purinosome formation to enhance DNPB while purine repletion leads to purinosome dissembly (9). Thus, purinosome disassembly is part of the mechanism by which purine salvage inhibits DNPB. Although several regulators of purinosome assembly have been identified (1, 12–14), less is known about purinosome disassembly during purine salvage.

Our search for genes that function in purine homeostasis identified *NUDT5*, which encodes the adenosine diphosphate (ADP)-ribose (ADPR) hydrolase Nudix Hydrolase 5, as a negative regulator of DNPB during purine salvage. NUDT5’s catalytic activity was dispensable for this effect. Instead, NUDT5 interacted with phosphoribosyl pyrophosphate amidotransferase (PPAT), the rate-limiting enzyme in DNPB, altering PPAT’s oligomeric state, reducing its enzymatic activity and accelerating purinosome disassembly during purine salvage.

## Ablation of NUDT5 confers cellular resistance to thiopurines.

**Figure 1.**
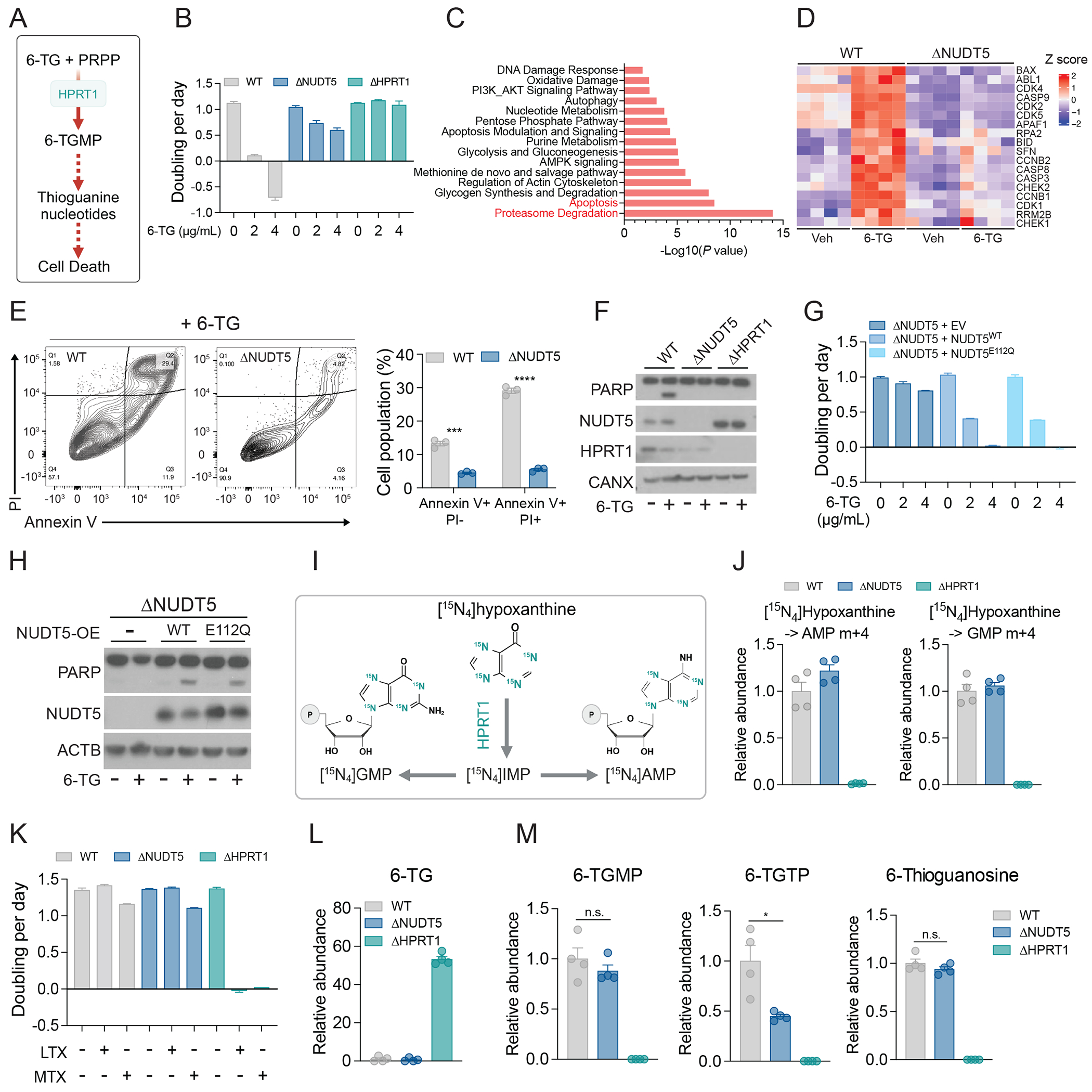
NUDT5 and HPRT1 regulate thiopurine sensitivity through different mechanisms. **(A)** Simplified schematic illustrating 6-TG metabolism-mediated cell death. **(B)** Growth rates of WT, ΔNUDT5, and ΔHPRT1 HeLa cells treated with the indicated doses of 6-TG. Data are technical replicates from one of three independent experiments. **(C)** Proteomics analysis showing upregulated pathways in 6-TG-treated WT cells compared to vehicle-treated WT, vehicle-treated ΔNUDT5 and 6-TG-treated ΔNUDT5 cells. **(D)** Heatmap showing abundance of proteins in the apoptosis pathway in WT and ΔNUDT5 HeLa cells treated with or without 0.5 μg/mL 6-TG for 24 hours (n=4). **(E)** Apoptosis analysis in WT and ΔNUDT5 HeLa cells treated with 0.5 μg/mL 6-TG for 48 hours. To the left are representative contour plots. To the right are bar graphs showing the percentage of apoptotic (Annexin V+, PI−) and dead (Annexin V+, PI+) cells. (n=3). **(F)** Western blot assessing cleaved PARP in WT, ΔNUDT5, and ΔHPRT1 HeLa cells treated with 0.5 μg/mL 6-TG for 24 hours. Calnexin (CANX) is the loading control. **(G)** Growth rates of ΔNUDT5 HeLa cells that express empty vector (EV), NUDT5^WT^, or NUDT5^E112Q^ treated with the indicated doses of 6-TG. Data are technical replicates from one of three independent experiments. **(H)** Western blot assessing cleaved PARP in ΔNUDT5 HeLa cells that express empty vector (EV), NUDT5^WT^, or NUDT5^E112Q^ treated with or without 0.5 μg/mL 6-TG for 24 hours. β-actin (ACTB) is the loading control. **(I)** Schematic illustrating labeling of purine nucleotides from [^15^N_4_]hypoxanthine. **(J)** Relative abundance of m+4 AMP and m+4 GMP from [^15^N_4_]hypoxanthine in WT, ΔNUDT5, and ΔHPRT1 HeLa cells during 4 hours of tracing. (n=4). **(K)** Growth rates of WT, ΔNUDT5, and ΔHPRT1 HeLa cells treated with DMSO, 1 μM lometrexol (LTX), or 1 μM methotrexate (MTX). Data are technical replicates from one of three independent experiments. **(L-M)** Relative abundance of 6-TG (L), 6-T-GMP, 6-T-GTP, and 6-thioguanosine (M) in WT, ΔNUDT5, and ΔHPRT1 HeLa cells treated with 0.5 μg/mL 6-TG for 24 hours. (n=4). Data points in each panel represent an independent sample unless specified. Error bars denote SEM. Multiple t test (E) and unpaired, two-sided t tests (M) were used for statistical analyses. ***: P < 0.001; ***: P < 0.001; *: P < 0.05; n.s.: P > 0.05. BioRender was used to generate the illustrations in (A and I).

To identify regulators of purine metabolism, we explored a published CRISPR screen for modifiers of 6-thioguanine (6-TG) sensitivity (15). 6-TG is converted to thioguanine nucleotides such as 6-thio-GMP (6-TGMP) by the salvage enzyme hypoxanthine-guanine phosphoribosyltransferase-1 (HPRT1). These nucleotides are incorporated into the genome, causing DNA damage and cell death (Fig. 1A). Guide RNAs targeting *HPRT1* and *NUDT5* were positively selected in 6-TG-treated cells (fig. S1A). Hydrolysis of ADPR by NUDT5 generates ribose-5-phosphate (R5P)(16). Because R5P is the precursor of PRPP, a substrate of HPRT1, NUDT5 has a plausible role in 6-TG toxicity by supporting salvage. To validate the screening result, we used CRISPR-Cas9 to delete *HPRT1* or *NUDT5* (ΔHPRT1, ΔNUDT5) in human HeLa cervical carcinoma cells and human A549 lung adenocarcinoma cells (fig. S1B). Loss of NUDT5 or HPRT1 increased the level of their substrates ADPR and hypoxanthine, respectively (fig. S1C and D), and conferred resistance to 6-TG and another thiopurine, 6-mercaptopurine (6-MP) (Fig. 1B and fig. S1E to G).

To assess how cells respond to 6-TG in the presence and absence of NUDT5, we performed quantitative proteomics on wild-type (WT) and ΔNUDT5 cells treated with solute or 6-TG (0.5 μg/mL). Pathway analysis revealed changes in protein abundance characteristic of apoptosis and proteasome degradation in WT but not ΔNUDT5 cells (Fig. 1C and D and table S1). Annexin V and propidium iodide (PI) staining confirmed a greater increase in apoptosis after thiopurine treatment in WT than ΔNUDT5 cells (Fig. 1E and fig. S1H). During thiopurine treatment, WT cells also exhibited more cleaved Poly(ADP-ribose) polymerase (PARP), another apoptosis maker, than ΔNUDT5 and ΔHPRT1cells. (Fig. 1F and fig. S1I). Conversely, overexpressing NUDT5 increased 6-TG-induced PARP cleavage (fig. S1J).

## NUDT5 regulates cellular responses to thiopurines independently of its catalytic function.

To test whether NUDT5’s role in thiopurine sensitivity is to provide R5P and PRPP, we rescued ΔNUDT5 cells with WT NUDT5 (NUDT5^WT^) or a NUDT5 mutant (NUDT5^E112Q^) lacking catalytic activity (17, 18). NUDT5^WT^, but not an empty vector or NUDT5^E112Q^, diminished ADPR abundance, confirming NUDT5^E112Q^,s catalytic defect (fig. S1K). However, both NUDT5^WT^ and NUDT5^E112Q^ restored sensitivity to thiopurines (Fig. 1G and H, fig. S1L and M), indicating that NUDT5 regulates thiopurine sensitivity independently of its catalytic function.

## Thiopurines suppress mitochondrial metabolism in a NUDT5-dependent manner.

Several pathways related to mitochondrial metabolism were suppressed in 6-TG-treated WT cells (fig. S2A and table S1). Mitochondrial electron transport chain (ETC) subunits and tricarboxylic acid (TCA) cycle enzymes were depleted in WT but not ΔNUDT5 cells treated with 6-TG (fig. S2B), as reported in acute lymphoblastic leukemia (ALL) cells (19). In parental cells, 6-TG decreased oxygen consumption, reduced contributions of oxidative glucose and glutamine metabolism to the TCA cycle, and enhanced the contribution of glutamine-dependent reductive carboxylation, a consequence of ETC suppression (20–22) (fig. S2C and S3A to D). All of these alterations were attenuated in ΔNUDT5 cells, indicating that 6-TG’s effects on mitochondrial function were NUDT5-dependent.

## NUDT5 and HPRT1 regulate cellular thiopurine sensitivity differently.

To examine NUDT5’s role in purine salvage, we conducted stable isotope tracing with [^15^N_4_]hypoxanthine. HPRT1 transfers four ^15^N nuclei from hypoxanthine into purine nucleotides, which are detected as m+4 isotopologues (Fig. 1I). Depletion of HPRT1 eliminated labeling from [^15^N_4_]hypoxanthine (Fig. 1J and fig. S4A). However, WT and ΔNUDT5 cells contained comparable labeling, indicating NUDT5’s dispensability for purine salvage (Fig. 1J and fig. S4A). WT and ΔNUDT5 cells were less sensitive than ΔHPRT1 cells to the DNPB inhibitors lometrexol (LTX) and methotrexate (MTX) (Fig. 1K and fig. S4B), also indicating intact purine salvage despite NUDT5 depletion. Lysates from ΔNUDT5 cells expressing empty vector, NUDT5^WT^, or NUDT5^E112Q^ had similar HPRT1 enzymatic activity (Fig. S4C). Thus, NUDT5 ablation is unlikely to confer 6-TG resistance through blocking 6-TG conversion to 6-T-GMP. To confirm this, we measured the relevant metabolites after 6-TG treatment. Intracellular 6-TG was barely detectable in WT and ΔNUDT5 cells after 24 hours of 6-TG treatment (Fig. 1L and fig. S4D). This was not due to defective 6-TG uptake, but to its conversion to 6-TGMP, 6-TGTP and 6-thioguanosine (Fig. 1M and fig. S4E). In contrast, ΔHPRT1 cells accumulated 6-TG but did not produce 6-TGMP, 6-TGTP, or 6-thioguanosine (Fig. 1L and M and fig. S4D and E). These data indicate that NUDT5 supports thiopurine toxicity through a mechanism distinct from HPRT1-mediated purine salvage.

## NUDT5 is dispensable for 6-TG-induced DNA damage.

Thiopurine incorporation into the genome causes DNA breaks (23–25). To investigate whether NUDT5 loss mitigated DNA damage during 6-TG treatment, we stained cells for phosphorylated H2A histone family member X (γH2AX), a nuclear DNA damage marker. WT and ΔNUDT5 cells had comparable numbers of γH2AX foci after 48-hours of 6-TG treatment (fig. S4F and G). Additionally, NUDT5 ablation did not protect cells against ionizing radiation (IR) or the DNA damaging agents, cisplatin and etoposide (fig. S5A to C). Although DNA mismatch repair (MMR) deficiency renders colorectal cancer cells resistant to 6-TG (23, 26), sgRNAs against MMR genes did not protect against 6-TG in the CRISPR screen (fig. S6A). To rule out a role for MMR, we deleted the gene encoding mutator gene L (MutL) protein homolog 1 (MLH1), a key MMR protein (fig. S6B). MLH1 depletion did not protect against 6-TG (fig. S6C). Therefore, NUDT5 specifically regulates sensitivity to thiopurines.

## Thiopurines suppress DNPB in a NUDT5-dependent fashion.

**Figure 2.**
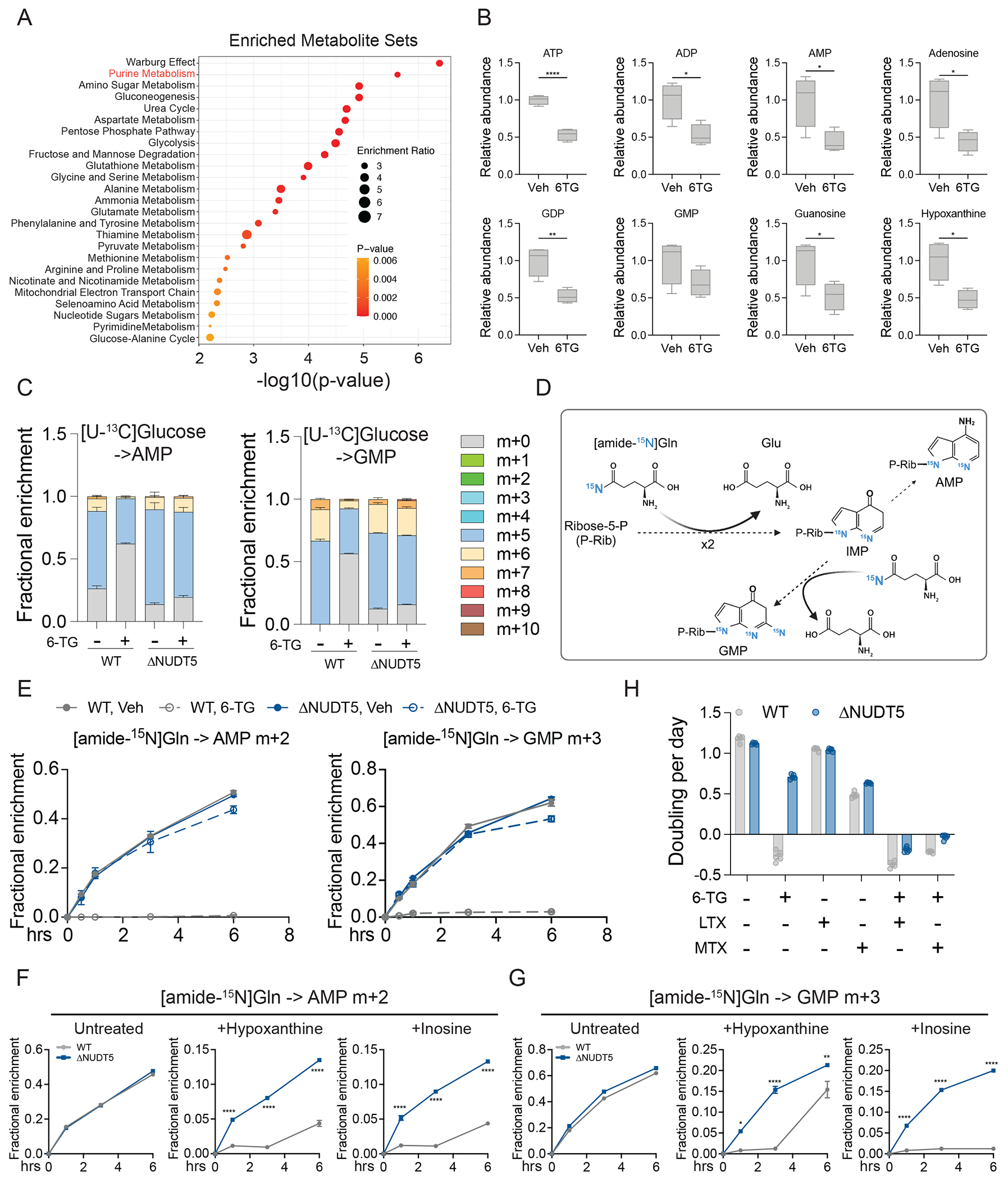
NUDT5 ablation overcomes thiopurine-induced blockade in de novo purine nucleotide synthesis. **(A)** Metabolite set enrichment analysis comparing vehicle and 24 hours of 0.5 μg/mL 6-TG-treated HeLa cells. **(B)** Relative abundance of the indicated purine metabolites in vehicle or 0.5 μg/mL 6-TG-treated HeLa cells. (n=4). **(C)** ^13^C labeling in AMP and GMP after 6 hours of culture with [U-^13^C]glucose in WT and ΔNUDT5 HeLa cells pre-treated with vehicle or 0.5 μg/mL 6-TG for 24 hours. (n=3). **(D)** Schematic illustrating labeling of purine nucleotides from [amide-^15^N]glutamine. **(E)** Time-dependent fractional enrichment of m+2 AMP and m+3 GMP from [amide-^15^N]glutamine in WT and ΔNUDT5 HeLa cells pre-treated with 0.5 μg/mL 6-TG for 24 hours. (n=3). **(F-G)** Time-dependent fractional enrichment of m+2 AMP (F) and m+3 GMP (G) from [amide-^15^N]glutamine in WT and ΔNUDT5 HeLa cells pre-treated with or without 20 μM hypoxanthine or inosine for 24 hours. (n=3). **(H)** Growth rates of WT and ΔNUDT5 HeLa cells treated with or without 4 μg/mL 6-TG, 1 μM LTX, 1 μM MTX or combinations. Data are technical replicates from one of three independent experiments. Data points in each panel represent an independent sample unless specified. Error bars denote SEM. Unpaired, two-sided t test (B) and two-way ANOVA (F and G) were used for the statistical analyses ****: P < 0.0001; **: P < 0.01; *: P < 0.05. BioRender was used to generate the illustration in (D).

Thiopurine-induced cell death may stem from DNPB suppression, because thiopurine nucleotides inhibit PPAT (1, 27). 6-TG elicited widespread metabolic changes, including suppression of many purine metabolites, in cells expressing NUDT5 (Fig. 2A and B, S7A, and table S2). Isotope tracing with [U-^13^C]glucose revealed that 6-TG suppressed purine nucleotide labeling in a pattern indicating decreased DNPB (Fig. 2C and fig. S7B and C). However, ΔNUDT5 cells did not display these changes (Fig. 2C and S7C). Suppressed DNPB in WT cells did not result from decreased synthesis of R5P, which was highly labeled under all conditions (Fig. S7D). Instead, 6-TG induced an accumulation of labeled R5P and other pentose phosphate pathway metabolites (Fig. S7E to G).

We also cultured cells with [amide-^15^N]glutamine, which delivers ^15^N to purine nucleotides largely through DNPB (Fig. 2D). Thiopurines nearly abolished AMP and GMP labeling in WT but not ΔNUDT5 cells (Fig. 2E and S7H), indicating that thiopurines required NUDT5 to suppress DNPB. In the absence of 6-TG, DNPB was unaffected by knockout or overexpression of NUDT5 (Fig. 2E and fig. S7H and I). Although thiopurines can impair purine salvage (28), the doses we used slightly enhanced the contribution of purine salvage to AMP and GMP in WT cells (fig. S8A and B), possibly in compensation for DNPB inhibition.

To test if NUDT5’s role in DNPB suppression is restricted to thiopurines, we treated cells with hypoxanthine and inosine to stimulate salvage. Treatment with either hypoxanthine or inosine decreased DNPB as assessed by labeling with [amide-^15^N]glutamine, and this was alleviated by loss of NUDT5 (Fig. 2F and G).

To test whether persistent DNPB underlies thiopurine resistance in ΔNUDT5 cells, we treated with DNPB inhibitors. ΔNUDT5 cells largely tolerated thiopurines or DNPB inhibitors individually, but concomitant treatment eliminated growth (Fig. 2H and fig. S8C). Supplementing WT cells with hypoxanthine or inosine restored growth during 6-TG treatment, likely by competing with 6-TG for metabolism and providing a source of natural nucleotides to counteract genotoxcity (fig. S8D and E). Altogether these data indicate that 6-TG induces purine nucleotide insufficiency through NUDT5-mediated DNPB suppression.

## NUDT5 binds PPAT to suppress DNPB during purine salvage.

**Figure 3.**
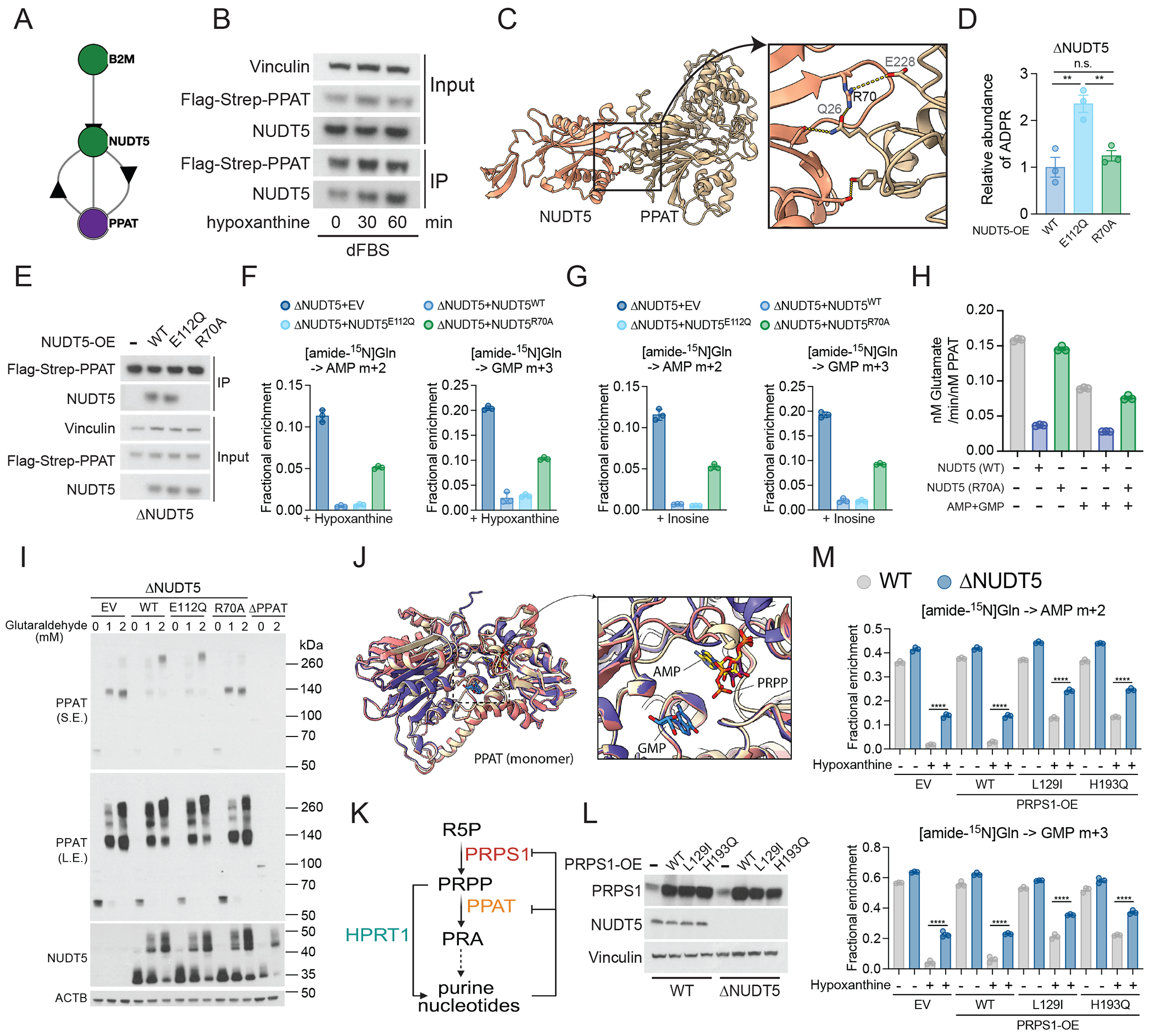
NUDT5 binds PPAT to suppress de novo purine biosynthesis during purine salvage. **(A)** Bio-plex data showing the interaction between PPAT and NUDT5. **(B)** Western blot showing the interaction between Flag-Strep-PPAT and NUDT5 in cells cultured in dialyzed FBS-supplemented medium after 20 μM hypoxanthine treatment at the indicated time points. Vinculin is the loading control for the input samples. **(C)** PPAT-NUDT5 interaction depicted by AlphaFold3. **(D)** Relative abundance of ADPR in ΔNUDT5 HeLa cells that express NUDT5^WT^, NUDT5^E112Q^, or NUDT5^R70A^. (n=3). **(E)** Western blot showing interaction between Flag-Strep-PPAT and NUDT5^WT^ or NUDT5^E112Q^ but not NUDT5^R70A^. Vinculin is the loading control for the input samples. **(F-G)** Fractional enrichment of m+2 AMP and m+3 GMP from [amide-^15^N]glutamine in ΔNUDT5 HeLa cells that express empty vector (EV), NUDT5^WT^, NUDT5^E112Q^, or NUDT5^R70A^ during 4 hours of tracing. The cells were treated with 20 μM hypoxanthine (F) or inosine (G) for 24 hours. (n=3). **(H)** Rates of PPAT-catalyzed synthesis of glutamate when incubated with or without NUDT5^WT^ or NUDT5^R70A^, AMP, and GMP. Data are technical replicates from one of three experiments. **(I)** Western blot showing oligomerization of PPAT and NUDT5 in ΔNUDT5 HeLa cells expressing empty vector (EV), NUDT5^WT^, NUDT5^E112Q^, or NUDT5^R70A^ and ΔPPAT cells. β-actin (ACTB) is the loading control. **(J)** PRPP, AMP, and GMP binding pockets in PPAT overlap as depicted by AlphFold3. **(K)** Schematic illustrating allosteric inhibition of PRPS1 and PPAT in the DNPB pathway. **(L)** Western blot showing overexpression of WT and mutant PRPS1 in WT and ΔNUDT5 cells. Vinculin is the loading control. **(M)** Fractional enrichment of AMP m+2 and GMP m+3 from [amide-^15^N]glutamine during 4 hours of tracing in WT or ΔNUDT5 cells that express empty vector (EV), PRPS1^WT^, PRPS1^L129I^, or PRPS1^H193Q^ with or without 24 hours of treatment with 20 μM hypoxanthine. (n=3). Data points in each panel represent an independent sample unless specified. Error bars denote SEM. One-way ANOVA (D and M) was used for the statistical analyses. ****: P < 0.0001; **: P < 0.01; *: P < 0.05. n.s.: P > 0.05. BioRender was used to generate the illustration in (K).

Two lines of evidence suggested that NUDT5 binds PPAT. First, the Biophysical Interactions of ORFeome-based Complexes database (BioPlex 3.0)(29, 30) reported such an interaction (Fig. 3A). Second, our in silico screen of human protein-protein interactions, which combines coevolution analysis and deep learning, predicted that NUDT5 and PPAT physically associate (31). We purified NUDT5 and the glutaminase domain of PPAT and used an in vitro binding assay to demonstrate that these proteins interact directly (fig. S9A to L). NUDT5 phosphorylation at threonine 45 (T45) increases stability of its dimeric form (18). To test if T45 phosphorylation regulates NUDT5’s interaction with PPAT, we generated T45 phosphomimetic (T45D) and T45 phospho-dead (T45A) mutants. Neither mutant affected NUDT5 binding to PPAT or thiopurine sensitivity (fig. S10A to E).

Supplementing the culture medium with hypoxanthine enhanced the PPAT-NUDT5 interaction in cells that had been cultured in purine-deprived medium (Fig. 3B). AlphaFold3 (32) predicted that NUDT5 arginine 70 (R70) resides in the interface between NUDT5 and PPAT and forms salt bridges with PPAT glutamine 26 (Q26) and glutamate 228 (E228) (Fig. 3C). We mutated R70 to alanine and reconstituted ΔNUDT5 cells with NUDT5^WT^, NUDT5^R70A^ or NUDT5^E112Q^. NUDT5^WT^ and NUDT5^R70A^, but not NUDT5^E112Q^, reduced cellular ADPR abundance, indicating that NUDT5^R70A^ is catalytically active (Fig. 3D). Epitope-tagged PPAT immunoprecipitated NUDT5^WT^ and NUDT5^E112Q^, but not NUDT5^R70A^, confirming that R70 is required for association with PPAT (Fig. 3E). NUDT5^R70A^ also mitigated DNPB suppression by hypoxanthine or inosine (Fig. 3F and G).

## NUDT5 binding suppresses PPAT enzymatic activity.

We next tested whether NUDT5 binding modulates PPAT enzymatic activity. We first generated PPAT-null cells (ΔPPAT) that express PPAT under control of doxycycline (fig. S11A). In the absence of doxycycline, these cells required purine bases or nucleosides to grow, consistent with DNPB blockade (fig. S11B and C). Lysates of cells expressing NUDT5^WT^ or NUDT5^E112Q^ had no detectable PPAT activity (i.e., same activity as ΔPPAT cells lacking doxycycline), whereas lysates without NUDT5 or with NUDT5^R70A^ exhibited higher activity (fig. S11D and E). Next, we purified full-length PPAT (fig. S11F and G), mixed it with purified NUDT5^WT^ or NUDT5^R70A^ in vitro and measured PPAT activity. NUDT5^WT^ but not NUDT5^R70A^ suppressed PPAT activity, with a magnitude of inhibition exceeding the effect of AMP and GMP at physiological concentrations (Fig. 3H).

## NUDT5 binding promotes PPAT oligomerization.

AMP and GMP promote PPAT tetramerization whereas the PPAT substrate PRPP keeps it in a dimeric state (33), indicating that dimeric PPAT is more enzymatically active. We explored whether NUDT5 impacts PPAT oligomerization. NUDT5^WT^ and NUDT5^E112Q^, but not NUDT5^R70A^, promoted PPAT oligomerization after glutaraldehyde crosslinking in cells (Fig. 3I; an AplhaFold3 rendering is in fig. S12A). Hypoxanthine modestly promoted PPAT oligomerization (fig. S12B and C), consistent with its positive effect on NUDT5-PPAT interaction. In cell lysates, PRPP is reported to disrupt this interaction, allowing PPAT to adopt its catalytically active dimeric form (33). Structural modeling predicts that the PPAT binding pockets for AMP and PRPP overlap with each other (Fig. 3J), allowing AMP to interfere with PRPP binding to PPAT. Therefore, during purine salvage, both the rapid consumption of PRPP by HPRT1 and the appearance of purine nucleotides may enhance NUDT5 binding to PPAT, promoting PPAT oligomerization and suppressing its enzymatic activity.

Purine nucleotides also allosterically inhibit PRPS1, which produces PRPP (34) (Fig. 3K). To discriminate between the effects of PRPS1 inhibition and NUDT5 on DNPB, we generated cells overexpressing PRPS1^L129I^ or PRPS1^H193Q^, mutant alleles from patients with PRPS1 superactivity syndrome (34) (Fig. 3L). These mutants partially resist allosteric inhibition by purine nucleotides and display higher levels of DNPB during hypoxanthine treatment (Fig. 3M). Deletion of NUDT5 in cells expressing mutant PRPS1 alleles further enhanced DNPB (Fig. 3M). These data imply that both PRPS1 inactivation and PPAT inhibition by NUDT5 contribute to DNPB suppression during purine salvage.

To explore mechanisms by which NUDT5 might regulate PPAT, we used the standalone version of AlphaFold3 to superimpose structures of PPAT bound to AMP, GMP or PRPP, and found that NUDT5 is not predicted to induce conformational changes in these binding pockets (fig. S13A–C)(32). PPAT catalyzes two reactions: a glutaminase step to remove ammonia from glutamine and a phosphoribosyltransferase (PRTase) step to conjugate the ammonia to PRPP, producing 5-phosphoribosyl-1-amine (PRA). These reactions are coordinated to ensure efficient delivery of ammonia to PRPP (35, 36). It is possible that in the PRPP-bound state, dimeric PPAT is more effective than tetrameric PPAT at gripping glutamine and facilitating ammonia channeling to the PRPase domain. Therefore, promotion of PPAT oligomerization by NUDT5 binding is potentially linked to the suppressed enzymatic activity.

## NUDT5 binds PPAT to stimulate purinosome disassembly.

**Figure 4.**
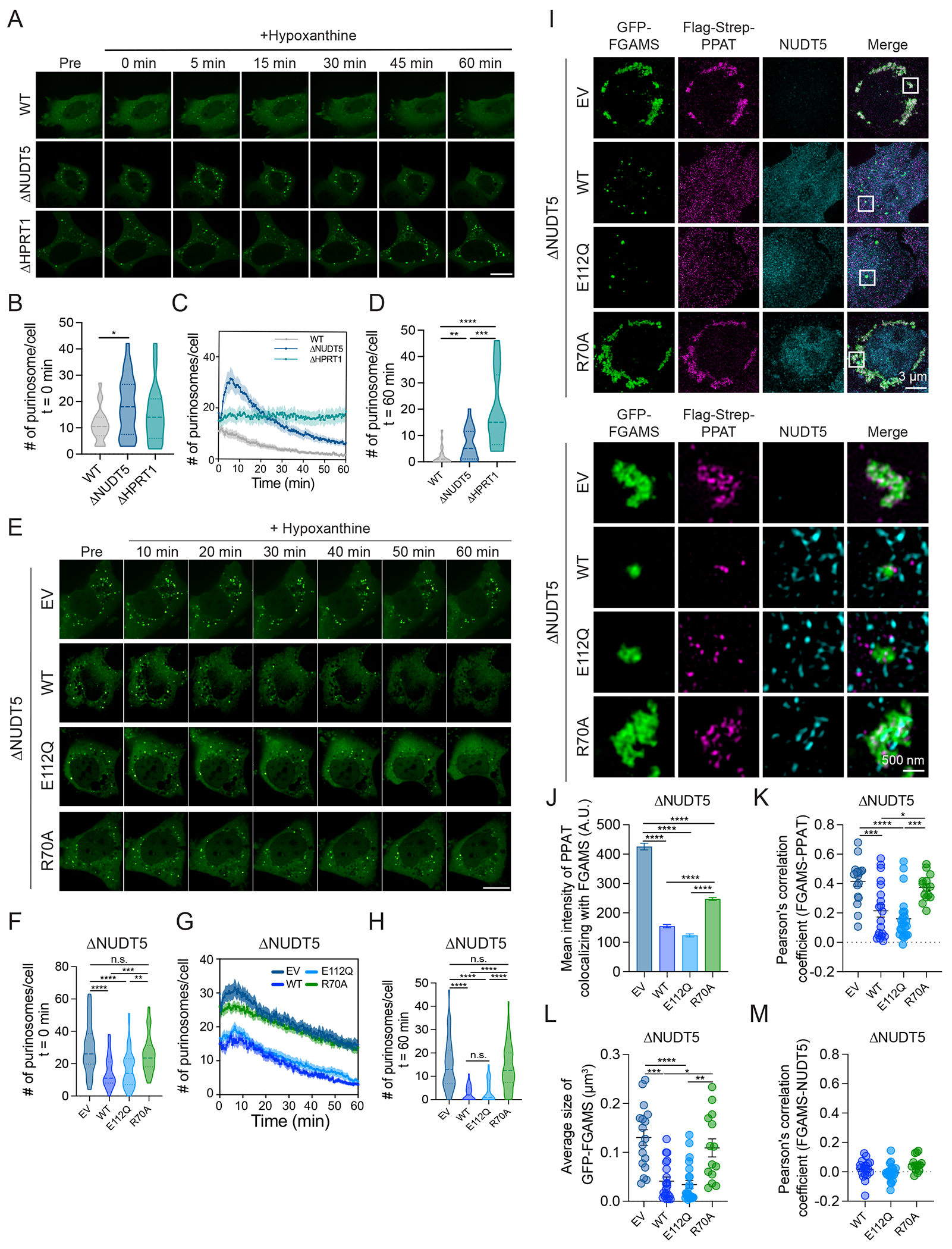
NUDT5 regulates purinosome dynamics. **(A)** Time-lapse imaging showing purinosome disassembly upon treatment with 20 μM hypoxanthine at t=0 minute in WT, ΔNUDT5 and ΔHPRT1 HeLa cells. The puncta indicate GFP-tagged FGAMS. The scale bar represents 10 μm. **(B-D)** Quantification of purinosome count at t=0 minute (B), over one hour (C), and at t=60 minutes (D) after hypoxanthine supplementation in WT (n=22), ΔNUDT5 (n=25) and ΔHPRT1 (n=18) HeLa cells. **(E)** Time-lapse imaging showing dispersal of purinosomes after treatment with 20 μM hypoxanthine at t=0 minute in ΔNUDT5 HeLa cells expressing an empty vector (EV), NUDT5^WT^, NUDT5^E112Q^, or NUDT5^R70A^. The puncta indicate GFP-tagged FGAMS. The scale bar represents 10 μm. **(F-H)** Quantification of purinosome count at t=0 minute (F), over one hour (G), and at t=60 minutes (H) after hypoxanthine supplementation in ΔNUDT5 HeLa cells expressing empty vector (EV) (n=42), NUDT5^WT^ (n=39), NUDT5^E112Q^ (n=47), or NUDT5^R70A^ (n=48). **(I)** Fluorescent images showing GFP-FGAMs (Green), Flag-strep-PPAT (Magenta), and NUDT5 (Cyan) in ΔNUDT5 HeLa cells expressing empty vector (EV), NUDT5^WT^, NUDT5^E112Q^, or NUDT5^R70A^ when cultured in purine-deprived medium. The bottom images are insets from the top images. (**J**) Mean intensity of Flag-Strep-PPAT colocalizing to GFP-FGAMS in (I) (n > 3000 for each group). (**K**) Pearson’s correlation coefficient of Flag-Strep-PPAT and GFP-FGAMS co-localization in ΔNUDT5 HeLa cells expressing EV (n=17), NUDT5^WT^ (n=20), NUDT5^E112Q^ (n=23), or NUDT5^R70A^ (n=14) in (I). (**L**) Size of GFP-FGAMS puncta in ΔNUDT5 HeLa cells expressing EV (n=17), NUDT5^WT^ (n=20), NUDT5^E112Q^ (n=23), or NUDT5^R70A^ (n=14) in (I). (**M**) Pearson’s correlation coefficient of NUDT5 and GFP-FGAMS co-localization in ΔNUDT5 HeLa cells expressing NUDT5^WT^ (n=19), NUDT5^E112Q^ (n=23), or NUDT5^R70A^ (n=14) in (I). Error bars denote SEM. One-way ANOVA (B, D, F, H, J, K, and L) was used for statistical analyses. ****: P < 0.0001; ***: P < 0.001; **: P < 0.01; *: P < 0.05. n.s.: P > 0.05.

PRA is labile, with a half-life of just a few seconds, and this poses a challenge to maintaining efficient DNPB (37). Cells cope with this by assembling PPAT and the other DNPB enzymes into cytosolic complexes called purinosomes, which channel intermediates from PRPP to inosine monophosphate (IMP) (9–11, 13, 37). Purinosomes arise during purine deprivation when the need for DNPB is high, then quickly disassemble during purine supplementation (9). To test if NUDT5 modulates purinosome dynamics, we expressed green fluorescent protein (GFP)-tagged phosphoribosylformyl glycinamidine synthase (FGAMS), a core purinosome protein, in WT, ΔNUDT5, and ΔHPRT1 cells. These cell lines were cultured in purine-depleted medium to induce purinosome formation, indicated by clustering of FGAMS-GFP into punctate structures (9, 12, 13) (Fig. 4A). NUDT5 deficiency modestly increased basal purinosome abundance (Fig. 4B). After supplementation with hypoxanthine, most punctate FGAMS-GFP structures disappeared in WT cells within one hour (Fig. 4A to D, Movie 1). Hypoxanthine had no effect on punctate FGAMS-GFP in ΔHPRT1 cells (Fig. 4A to D, Movie 2). In ΔNUDT5 cells, hypoxanthine acutely stimulated punctate FGAMS-GFP, then delayed dispersal, resulting in persistent punctate signals after one hour (Fig. 4A to D, Movie 3). In ΔNUDT5 cells reconstituted with NUDT5, hypoxanthine-induced FGAMS-GFP dispersal was accelerated by NUDT5^WT^ and NUDT5^E112Q^, but not NUDT5^R70A^ (Fig. 4E to H, Movies 4–7). Super-resolution 3D structured illumination microscopy revealed that NUDT5^WT^ and NUDT5^E112Q^, but not NUDT5^R70A^, impeded PPAT’s interaction with FGAMS and reduced the size of FGAMS-GFP without being physically partitioned into purinosomes (Fig. 4I to M). Most NUDT5 was not colocalized with PPAT, suggesting that these proteins interact transiently in intact cells (Fig. 4I). Together, the data show that NUDT5 negatively regulates purinosome formation and facilitates purinosome disassembly during purine salvage.

## Disrupting the PPAT-NUDT5 interaction confers resistance to thiopurines.

**Figure 5.**
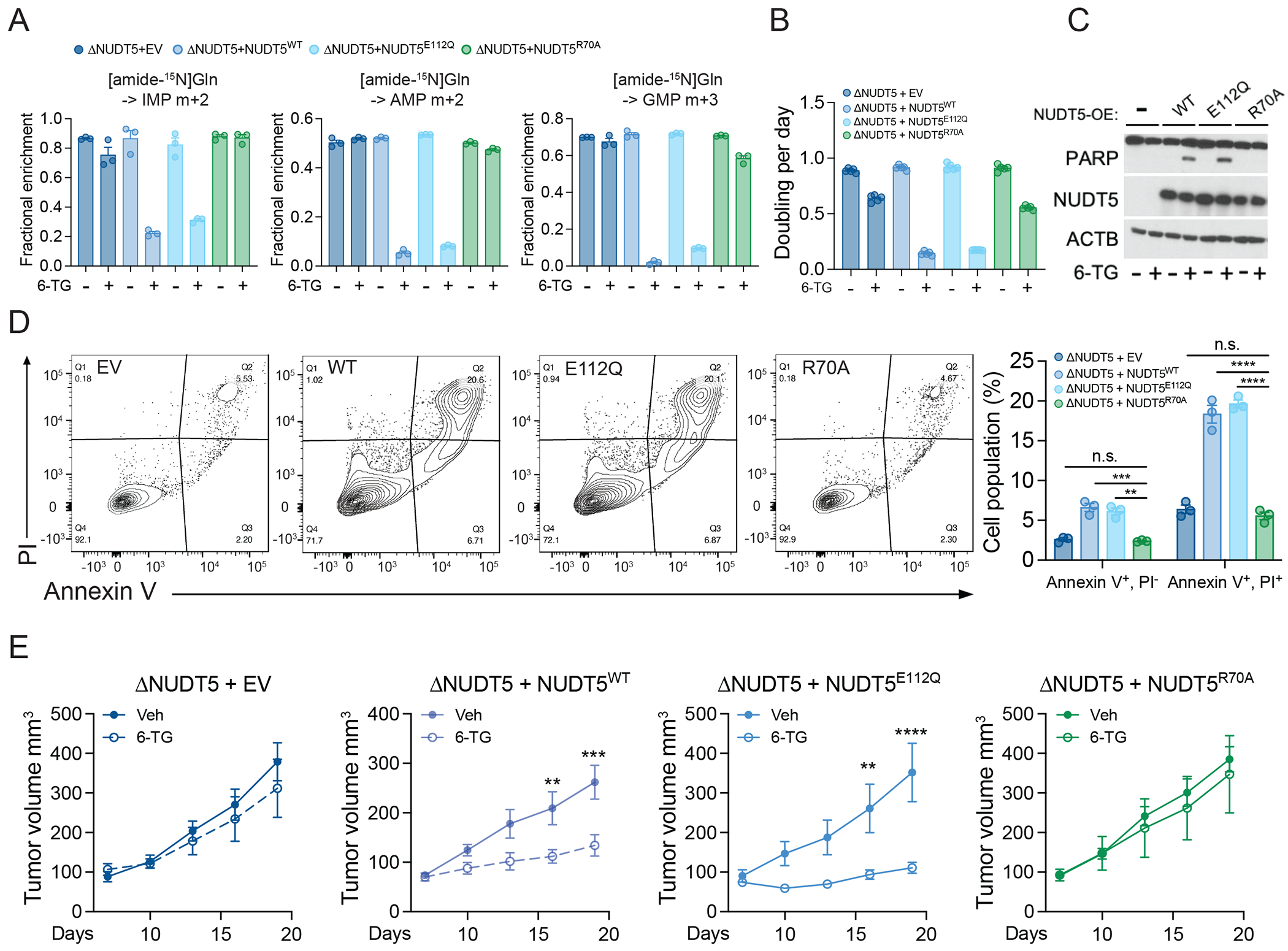
Disruption of the NUDT5-PPAT interaction induces thiopurine resistance. **(A)** Fractional enrichment of m+2 IMP, m+2 AMP and m+3 GMP from [amide-^15^N]glutamine in ΔNUDT5 HeLa cells that express empty vector (EV), NUDT5^WT^, NUDT5^E112Q^, or NUDT5^R70A^ during 6 hours of tracing. The cells were pre-treated with 0.5 μg/mL 6-TG for 24 hours. (n=3). **(B)** Growth rates of ΔNUDT5 HeLa cells that express empty vector (EV), NUDT5^WT^, NUDT5^E112Q^, or NUDT5^R70A^, treated with 2 μg/mL 6-TG. Data are technical replicates from one of three experiments. **(C)** Western blot assessing cleaved PARP in ΔNUDT5 HeLa cells that express empty vector (EV), NUDT5^WT^, NUDT5^E112Q^, or NUDT5^R70A^, treated with 0.5 μg/mL 6-TG for 24 hours. β-actin (ACTB) is the loading control. **(D)** Apoptosis analysis in ΔNUDT5 HeLa cells that express empty vector (EV), NUDT5^WT^, NUDT5^E112Q^, or NUDT5^R70A^, treated with 0.5 μg/mL 6-TG for 48 hours. To the left are representative contour plots. To the right are bar graphs showing percentage of apoptotic (Annexin V+, PI−) and dead (Annexin V+, PI+) cells. (n=3). (**E**) Subcutaneous growth of ΔNUDT5 HeLa xenografts expressing empty vector (EV), NUDT5^WT^, NUDT5^E112Q^, or NUDT5^R70A^ treated with Vehicle (Veh) or 2 mg/kg 6-TG. EV, Veh (n=8); EV, 6-TG (n=6); NUDT5^WT^, Veh (n=8); NUDT5^WT^, 6-TG (n=8); NUDT5^E112Q^, Veh (n=8); NUDT5^E112Q^, 6-TG (n=8); NUDT5^R70A^, Veh (n=8); NUDT5^R70A^, 6-TG (n=8). Data points in each panel represent an independent sample unless specified. Error bars denote SEM. Two-way ANOVA was used were used for statistical analysis (D and E). ****: P < 0.0001; ***: P < 0.001; **: P < 0.01; n.s.: P > 0.05. Error bars denote SEM.

Cells expressing NUDT5^WT^ or NUDT5^E112Q^ suppressed DNPB upon exposure to thiopurines, whereas cells expressing NUDT5^R70A^ had comparable DNPB under these conditions (Fig. 5A and fig. S14A). Because the PPAT-NUDT5 interaction enabled thiopurine-induced DNPB suppression, we reasoned that loss of the interaction would allow cells to survive thiopurine treatment. Indeed, compared to cells expressing either NUDT5^WT^ or NUDT5^E112Q^, cells expressing NUDT5^R70A^ maintained growth and resisted apoptosis when treated with thiopurines, similar to ΔNUDT5 cells (Fig. 5 B–D and fig. S14B). Xenografts lacking NUDT5 or expressing NUDT5^R70A^ were also resistant to 6-TG treatment, whereas xenografts expressing NUDT5^WT^ or NUDT5^E112Q^ were sensitive (Fig. 5E).

## Discussion

NUDT5 regulates DNPB through two complementary mechanisms: suppression of PPAT enzymatic activity and enabling purinosome disassembly (fig. S14C and D). Neither effect requires NUDT5’s catalytic activity but both require its association with PPAT. The processes governing purinosome dynamics are incompletely understood, but NUDT5’s involvement suggests that disassembly is prompted by PPAT oligomerization. Presumably purinosome disassembly also involves a metabolic signal, but the identity of this signal is unknown. Hypoxanthine prompts purinosome disassembly over time, but this requires both HPRT1 and the transient association of NUDT5 with PPAT. Potential metabolic triggers in this context include increased purine nucleotides, decreased PRPP, or the ability to biochemically inhibit PPAT. When NUDT5 cannot associate with PPAT, hypoxanthine paradoxically induces a transient increase in purinosomes, implying that decisive purinosome disassembly requires rapid suppression of PPAT.

DNPB inhibition activates purine salvage and vice versa, reflecting plasticity in how cells maintain an adequate purine nucleotide supply (3, 8). NUDT5 prevents simultaneous DNPB and salvage. This is likely advantageous because purine overabundance is pathological. Increased purine:pyrimidine ratios induce DNA replication stress and cell cycle arrest (5), and excess purine synthesis causes hyperuricemia and associated toxicities. NUDT5 facilitates purine homeostasis by allowing cells to switch off DNPB during salvage.

Thiopurines are used to treat ALL and inflammatory diseases (38–40). Genetic variants affecting the metabolism of thiopurines to thiopurine nucleotides impact both clinical efficacy and the substantial systemic toxicities associated with these drugs (40–44). Patients with *HPRT1* variants that impair purine salvage are refractory to thiopurines (43), whereas loss-of-function variants in Nudix Hydrolase 15 (*NUDT15*), an enzyme involved in thioguanine nucleotide detoxification, increase sensitivity (42, 45). The human relevance of NUDT5’s role in thiopurine sensitivity, as shown here and in other recent reports (46–48), is underscored by the fact that genetic variants promoting *NUDT5* expression are associated with enhanced sensitivity to thiopurine treatment in patients (49).

## Supplementary Material

Supplemental Material

Table S1

Table S2

Source data

## Figures and Tables

**Movie_1:** 

**Movie_2:** 

**Movie_3:** 

**Movie_4:** 

**Movie_5:** 

**Movie_6:** 

**Movie_7:** 

## Data Availability

Cell lines and plasmids generated for this study are available under an MTA. All data are available in the manuscript or supplementary materials. Source data are in table S3. Raw proteomics data have been deposited in MassIVE (accession number: MSV000097432). The codes used for the imaging data analyses in this paper are in codes S1 and S2. The codes used for the proteomics analysis in this paper have been deposited in GitHub (https://github.com/cailing20/Wu_et_al_2025_proteomics).
