# Supplementary material for: NUDT5 regulates purine metabolism and thiopurine sensitivity by interacting with PPAT
Source: Science. Author manuscript; Available in PMC 2026 Jan 30. (PMC12853130; doi:10.1126/science.adx9717)
Supplement: Supplemental Material [file NIHMS2134690-supplement-Supplemental_Material.pdf]

Science
AAAS

Supplementary Materials for

NUDT5 regulates purine metabolism and thiopurine sensitivity by interacting with PPAT

Zheng Wu, Phong T Nguyen, Varun Sondhi, Run-Wen Yao, Zhifang Lu, Tao Dai, Jui-Chung Chiang, Feng Cai, Imani M Williams, Eliot B Blatt, Zengfu Shang, Ling Cai, Jing Zhang, Mya D Moore, Islam Alshamleh, Xiangyi Li, Tamaratare Ogu, Lauren G Zacharias, Rainah Winston, Joao S Patricio, Xandria Johnson, Wei-Min Chen, Qian Cong, Thomas P Mathews, Yuanyuan Zhang, Limei Zhang, Ralph J DeBerardinis

Correspondence to: Ralph.DeBerardinis@utsouthwestern.edu

This PDF file includes:

Materials and Methods
Figs. S1 to S14
Captions for Data S1 to S14
Captions for Movies 1 to 7
References (50-60)

Other Supplementary Materials for this manuscript include the following:

Movies 1 to 7
Tables S1 to S3
Codes S1 and S2 (.ijm files)
MDAR Reproducibility Checklist

Materials and Methods

Cell culture

HeLa cells were a gift from Dr. Javier Garcia-Bermudez's laboratory at University of Texas Southwestern. A549 (CCL-185, ATCC) and HEK293T (CRL-3216, ATCC) cells were purchased from American Type Culture Collection (ATCC). HeLa and A549 cells were cultured in RPMI-1640 (R8758, Sigma-Aldrich) supplemented with 10% fetal bovine serum (100106, GeminiBio). HEK293T cells were cultured in Dulbecco's modified Eagle medium with high glucose (D5796, Sigma-Aldrich) supplemented with 10% fetal bovine serum. ∆PPAT HeLa cells were cultured in the presence of 50 µM inosine (57510, Sigma-Aldrich) or hypoxanthine (H9636, Sigma-Aldrich). HEK293F (R79007, Invitrogen) cells were cultured in Freestyle 293 expression medium (12-338-026, Thermo Fisher Scientific) at 37˚C, 8% CO_2, shaking at 130 rpm. All other cells were grown at 37˚C in a humidified atmosphere with 5% CO_2. Cells were routinely subjected to mycoplasma testing.

Gene deletion and over-expression

To generate *HPRT1* and *NUDT5* knockouts with a Cas9 D10A nickase system, cells were transfected with two modified pDG461 constructs (*50*) with mGreenLantern and mScarlett fluorescent markers. Each vector contained two sgRNAs against the indicated gene or scrambled controls. The cells were sorted for green fluorescent protein (GFP) and red fluorescent protein (RFP) double-positive populations 48 hours after transfection. ∆PPAT cells were generated by transfection of a construct that integrates at the Rogi1 safe harbor locus (*51*) and expresses gRNA-resistant PPAT and blue fluorescent proteins (BFP) under control of doxycycline. After transfection, cells were passaged for a week in the presence of 100 ng/mL doxycycline (BP26531, Fisher Scientific) before being sorted for BFP-positive populations. The BFP-positive cells were then transfected with modified pDG461 constructs containing sgRNAs against the endogenous PPAT followed by flow sorting for GFP and RFP-double positive cells. The sorted single cells were cultured in the presence of 100 ng/mL doxycycline. After clones were transferred into 6-well plates, doxycycline was removed and 50 µM hypoxanthine or inosine was supplemented. To generate cell lines that over-express WT or mutant NUDT5 and PRPS1, the cells were transfected with constructs that contain cDNAs of the genes of interest that integrated at the Rogi2 safe harbor locus (*51*). The cells were then selected by culturing with 1 µg/mL puromycin (Thermo Fisher Scientific, NC9138068) 48 hours after transfection until all non-transfected cells were dead. To generate sgScr and sgMLH1 HeLa cells, the indicated gRNAs were cloned into the LentiCRISPRv2 vector (*52*), a gift from Feng Zhang (Addgene plasmid # 52961; http://n2t.net/addgene:52961; RRID:Addgene_52961) followed by transfection into HEK293T cells using Lipofectamine 3000 (Thermo Fisher Scientific L3000015) at a ratio of 2:1 for psPAX2:pMD2G to generate lentivirus. To generate HeLa cells expressing streptavidin-binding Flag-Strep-PPAT, the cDNA was cloned into the PMXS-IRES-Bsd retroviral expression vector followed by transfection into 293T cells using Lipofectamine 3000 (Thermo Fisher Scientific L3000015) at a ratio of 2:1 for of Gag-Pol:VSVG to generate retrovirus. All virus-containing media were collected 48 hours after transfection and filtered through 0.45 µm membranes, then immediately added to HeLa cell cultures containing 4 µg/mL polybrene (Sigma, TR-1003-G). After 24 hours, 1 µg/mL puromycin and 10 µg/mL blasticidin (Thermo Fisher Scientific,

NC1366670) were used to select sgScr/sgMLH1 expressing cells and Flag-Strep-PPAT overexpressing cells, respectively, until all non-infected cells were dead. Both deletion and overexpression of the proteins were validated by western blot. DNA oligos were purchased from IDT. The gRNA sequences were:

sgRNA	Sequence
HPRT1 #1	5'- AGTCCTACAGAAATAAAATC-3'
HPRT1 #2	5'- GATGTGATGAAGGAGATGGG-3'
HPRT1 #3	5'- TGTGTGACACAGGCAGACTG -3'
HPRT1 #3	5'- GCATCACAACATTGACACTG -3'
NUDT5 #1	5'- AATGTGAACAGGGAGGCCCG -3'
NUDT5 #2	5'- GTTCCCTCACATACTGAGAC -3'
NUDT5 #3	5'- ACAGTATATCATTTCAGAGG -3'
NUDT5 #4	5'- CCATTCTGAGAAGATTCCGT -3'
PPAT #1	5'- TGATCACTCTGGGACTCGTG -3'
PPAT #2	5'- ATGCGGTACATCCAGCTGCG -3'
PPAT #3	5'- GGTTGCCTAGGCGTGCACGA -3'
PPAT #4	5' -GGTACTGGCTTAGGTCGGAG -3'
Scrambled (Scr)	5'- TTCTTAGAAGTTGCTCCACG -3'
sgMLH1	5'- TGATCCCGGTGCCATTGTCT -3'

Xenograft experiment

The mouse xenograft experiment was conducted in compliance with institutional guidelines and approved by the Institutional Animal Care and Use Committee at the University of Texas Southwestern Medical Center (protocol 2016-101694). HeLa cells were suspended in Opti-MEM medium (11058021, Invitrogen) and mixed 1:1 with Matrigel (CB-40234, Thermo Fisher Scientific). One million cells were subcutaneously injected into the right flank of 9-week-old female J:NU mice (RRID:IMSR_JAX:007850, Jackson Laboratory). One week later, the mice were randomized to receive daily intraperitoneal injections of vehicle or 2 mg/kg 6-TG in sterile saline. Tumor growth was monitored by measuring two orthogonal diameters, and tumor volumes were calculated using the formula $V = (L1x(L2)^2)/2$. Mice that died from 6-TG toxicity before termination of the experiment were excluded from analysis.

Quantitative proteomics

WT and ΔNUDT5 cells were treated with vehicle or 0.5 µg/mL 6-TG for 24 hours. The cells were rinsed twice with ice-cold PBS then lysed with a solution containing 5% SDS in 50 mM TEAB with protease and phosphatase inhibitors. Cells were scraped on ice and transferred to 1.5 mL Eppendorf tubes and incubated at room temperature for 15 minutes to allow complete lysis. Proteins were quantified using DC Protein Assay Kit (Bio-Rad, 5000111) and normalized to the same concentration. These samples were subjected to overnight digestion with trypsin using S-Trap (Protifi) after disulfide bond reduction and alkylation. The eluted peptide from the S-Trap was dried and reconstituted in 100 mM TEAB buffer. The samples were labeled using a TMT10plex Isobaric Mass Tagging Kit (Thermo) following the manufacturer's instructions. The combined sample was passed through solid-phase extraction using an Oasis HLB plate (Waters) and dried in a SpeedVac. The dried sample was reconstituted in 0.1% TFA buffer containing 2%

acetonitrile, and then diluted down to ~1 μg of peptides before injection. A Thermo Orbitrap Eclipse MS system coupled to an Ultimate 3000 RSLC-Nano liquid chromatography system was used to analyze the peptides in the UT Southwestern Proteomics Core Facility, as previously described (*8*).

To remove potential bias from differing sample intensities, normalization factors were calculated by summing abundances across non-missing entries and dividing by the mean total intensity. These normalization factors were used to scale each dataset accordingly. Statistical analyses were performed using a custom function based on linear modeling and t-tests. For each analyte, we assessed: 1) treatment effect within genotypes using Welch's t-test and Cohen's d effect size; 2) genotype effect under each treatment using similar t-tests; and 3) interaction effects using two-way ANOVA models of the form value ~ genotype * treatment. P-values were adjusted using the Benjamini–Hochberg method for false discovery rate (FDR) control. Enrichment of pathway terms was performed using a hypergeometric test to assess the overlap between significantly altered proteins in our dataset, selected by fold-change direction and adjusted p-value < 0.05), and curated pathway gene sets from public databases (Wikipathways). Z-scored expression values were used for heatmap visualization to compare selected features across genotypes, treatments, and replicates. Data were visualized using the ComplexHeatmap package (*53*).

Targeted metabolomics

To extract metabolites, the cells were rinsed in ice-cold saline and quenched with 80% acetonitrile. The cells were incubated at -80˚C for at least 20 minutes, then transferred onto ice, scraped and subjected to three freeze-thaw cycles between liquid nitrogen and a 37˚C water bath. Afterwards, the samples were vortexed for 1 minute and spun down at 4˚C at 20,160 x g for 15 minutes. The supernatants were collected for a second spin-down under the same conditions. Then the supernatants were transferred into fresh Eppendorf tubes followed by protein quantification and normalization prior to analysis on Thermo Exploris or Q-Exactive liquid chromatograph/mass spectrometry systems. Chromatographic separation of metabolites was performed using a Vanquish UHPLC system equipped with a ZIC-pHILIC column (Millipore-Sigma, Burlington, MA) as previously described (*8*). Extracted ion chromatograms (XICs) were produced with a mass tolerance of 5 ppm. Analyte identities were verified using purified standards and product ion spectra. MetaboAnalyst 6.0 was used to analyze the metabolomics data and generate the principal component analysis plot (*54*).

Gas chromatography/mass spectrometry (GC/MS)

To extract metabolites, the cells were rinsed with cold saline, scraped and subjected to three freeze-thaw cycles as above. After vortexing for 1 minute, the samples were centrifuged at 20,160 x g at 4˚C for 15 min. The supernatants were transferred to new Eppendorf tubes and dried in a SpeedVac concentrator overnight. The dried metabolites were re-suspended in 30 µL of anhydrous pyridine containing 10 mg/mL methoxyamine. After a short vortex and centrifugation, the supernatants were transferred to GC/MS autoinjector vials and incubated at 75˚C for 15 minutes. 70 µL N-(tert-butyldimethylsilyl)-N-methyltrifluoroacetamide (MTBSTFA) was added into each vial followed by incubation at 75˚C for 1 hour. 1 µL of each sample was injected onto an Agilent 5973N or 5975C Mass Spectrometer coupled to Agilent 6890 or 7890 gas chromatographs. EL-MAVEN

was used to analyze the data and MATLAB was used to correct for natural abundance as previously described (*55*).

Stable isotope tracing

For [U-^{13}C]glucose tracing, cells were cultured in glucose-free RPMI medium (Sigma, R1383-L) supplemented with 11 mM [U-^{13}C]glucose (Cambridge Isotope Laboratories, CLM481-0.25) and 10% dialyzed FBS (Gemini Bio-Products, 100108). For glutamine tracing, cells were cultured in glutamine-free RPMI medium (Sigma, R0883) supplemented with 2 mM [amide-^{15}N]glutamine (Cambridge Isotope Laboratories, NLM-557-1) or 2 mM [U-^{13}C]glutamine (Cambridge Isotope Laboratories, CLM-1822-0) and 10% dialyzed FBS. For [$^{15}N_4$]hypoxanthine tracing, cells were cultured in RPMI medium containing 10% dialyzed FBS and 10 μM [$^{15}N_4$]hypoxanthine (Cambridge Isotope Laboratories, NLM-8500-0.1). If cells were pretreated with any drugs, they were exposed to the same drugs during tracing. Tracing time points are indicated in the figure legend. Extracted metabolites were subjected to analyses with GC/MS or Q-Exactive MS as described above, or with an AB SCIEX QTRAP 5500 LC/triple quadrupole MS (Applied Biosystems SCIEX) as previously described (*8*).

HPRT1 enzymatic activity analysis

HPRT1 enzyme activity was measured using the Precise HPRT1 assay kit (Novo CIB, K0709-01-2) according to the manufacturer's instructions. Briefly, HeLa cells were cultured in 15 cm plates, washed once with PBS, collected by scraping, and lysed in ice-cold lysis buffer containing 150 mM NaCl, 10 mM Tris-HCl pH 7.4, 1 mM EDTA, and 1% NP-40. The lysates were centrifuged at 18,000 x g for 10 minutes at 4˚C. Protein concentrations were determined using the DC Protein Assay Kit, then the samples were diluted to the same concentration. Each enzymatic reaction included 5 μL of sample or positive control (human recombinant HPRT enzyme) and 100 μL of the reaction mixture containing NAD, DTT, and bacterial IMPDH. The reactions were performed either with or without 2 mM PRPP at 37˚C. Absorbance at 340 nm was measured every 2 minutes for 2 hours.

Cell doubling analysis

Cells were seeded in clear, flat 96 well plates the day before treatment. To count the cell number, cells were stained with 1 μg/mL Propidium Iodide (PI) (Thermo Fisher Scientific, P3566) and 5 μg/mL Hoechst (Thermo Fisher Scientific, 62249) in PBS at 37˚C for at least 15 minutes. The plates were then analyzed using a Celigo Imaging Cytometer. Live cell numbers were calculated by subtracting the PI-positive cells from the Hoechst-positive cells. The doubling rates were calculated as previously described (*56*).

Colony formation assay

Cells were plated in 60-mm dishes at a density ranging from 200 to 2000 cells per dish. Subsequently, the cells were subjected to the indicated doses of ionizing irradiation (0-6 Gy), with each treatment performed in triplicate. After an incubation period of 9 days, the colonies were fixed using 6% glutaraldehyde (G6257, Sigma-Aldrich) and stained with 0.5% crystal violet solution (C0775, Sigma-Aldrich). Colonies containing more than 50 cells were counted and quantified. The surviving fraction was then normalized to the corresponding sham control. Survival fraction curves were fitted using the linear-quadratic model by GraphPad Prism.

Immunoblotting

Immunoblotting was performed as previously described (*8*). In brief, RIPA buffer (Boston BioProducts, BP-115) containing proteinase and phosphatase inhibitors (Thermo Fisher Scientific, 78444) was used to lyse cells. Samples were spun down at 20,160 x g at 4˚C for 10 minutes followed by protein quantification using the DC Protein Assay Kit. Equal amounts of protein were loaded on the gel, followed by transfer to PVDF membranes (Thermo Fisher Scientific, 88518). Membranes were soaked in methanol for a few seconds and then rinsed with DI water prior to air drying. The dried membrane was incubated with primary antibodies diluted in filtered 5% BSA in PBS with 0.1% Tween-20 (PBST) overnight in the cold room. The membranes were washed with PBS three times for 5 minutes at room temperature followed by incubation with the secondary antibody (7074, Cell Signaling Technology, RRID:AB_2099233; 7076, Cell Signaling Technology, RRID:AB_330924; 31480, Thermo Fisher Scientific, RRID:AB_228457) diluted in 5% non-fat milk in PBST for 1 hour at room temperature. Membranes were washed three times for 5-10 minutes with PBS at room temperature before being incubated with Pierce ECL (PI32106, Thermo Fisher Scientific) for 2 minutes. Autoradiography films were used to detect the signals. Antibodies are: anti-PARP (9542s, Cell Signaling Technology, RRID:AB_2160739); anti-NUDT5 (AF6414-SP, R&D Systems, RRID:AB_3644397); anti-HPRT1 (sc-376938, Santa Cruz Biotechnology, RRID:AB_2938532); anti-Calnexin (ADI-SPA-860-F, Enzo Life Sciences, RRID:AB_11178981); anti-β-Actin (C6198, Sigma-Aldrich, RRID:AB_476856); anti-Flag (F3165, Sigma-Aldrich, RRID:AB_259529); anti-Vinculin (13901S, Cell Signaling Technology, RRID:AB_2728768), anti-PRPS1 (15549-1-AP, Proteintech, RRID:AB_10694269), and anti-PPAT (15401-1-AP, Proteintech, RRID:AB_2166532).

Co-immunoprecipitation

Flag-Strep-tagged PPAT and different variants of NUDT5 were over-expressed in ΔNUDT5 HeLa cells as described in **Gene deletion and over-expression**. Cells were scraped in cold PBS on ice and pelleted at 4˚C, before being lysed in a buffer containing 20 mM Tris-HCl pH 7.5, 150 mM NaCl, 1 mM EDTA, 1% NP-40, and protease inhibitors (1 µg/ml leupetin, 1 µg.ml pepstatin, 1 mM benazamidine HCl). The cell lysates were subjected to three freeze-thaw cycles between liquid nitrogen and a 37 ^{o}C water bath, followed by centrifugation at 18,000 x g for 15 minutes at 4 ^{o}C. The supernatants were collected for protein quantification using the DC protein assay kit. After protein normalization, 50 µL supernatants were collected as the input samples mixed with 6x Laemmli buffer and boiled at 95˚C for 5 minutes. At least 1 mg protein from the supernatant was incubated with 50 µL 50% Strep-Tactin XT Sepharose chromatography resin (Sigma, GE29401324) on a rotator at 4˚C for 2 hours. The samples were spun down at 1,000 x g at 4˚C for 30 seconds. The supernatant was aspirated and the resin was washed with 1 mL PBST followed by centrifugation at 1,000 x g at 4˚C for 30 seconds. After repeating the wash five times, 25 µL 2x Laemmli buffer was added followed by 5 minutes of incubation at 95˚C to elute the samples three times. Western blots were performed as described in **Immunoblotting.**

Seahorse XFe96 Respirometry

Oxygen consumption rates were measured using An XFe96 Extracellular Flux Analyzer (Agilent Technologies) as previously described (*8*). In brief, cells were treated with 0.5 µg/mL 6-TG in the morning. 6-8 hours later, cells were re-seeded in a 96-well Seahorse

plate with 20,000 cells per well while being exposed to the same concentration of 6-TG for 16-18 hours. Cells were then washed with Seahorse medium three times followed by at least 30 minutes of incubation in a CO_2-free incubator at 37˚C before the analysis. Oxygen consumption rates were normalized to the cell number in each well.

γH2AX foci analysis

Cells were seeded on coverslips prior to treatment with vehicle or 0.5 µg/mL 6-TG for 48 hours. Cells were fixed in 4% paraformaldehyde for 15 minutes followed by 10 minutes of permeabilization in 0.1% (v/v) Triton X-100 at room temperature. Cells were then blocked with 1% BSA in PBS for 30 min at room temperature prior to incubation with primary antibodies against γH2AX (1:500) (05-636, Millipore, RRID:AB_309864) at room temperature for 1 hour. Cells were washed with PBS three times before being incubated with secondary antibodies conjugated with fluorophores in the dark for 1 hour at room temperature. After 4 x 5 minutes of washing in PBS, the coverslips were mounted and sealed onto glass slides using antifade (P36935, Invitrogen) and nail polish, respectively. Fluorescent images were captured by Zeiss LSM 880 I laser Scanning Microscope. γH2AX foci were quantified using Image J.

Purinosome analysis

Live cell fluorescence microscopy analysis of purinosomes was conducted as previously described using transiently expressed FGAMS-GFP (Addgene plasmid # 99107; http://n2t.net/addgene:99107; RRID:Addgene_99107) as the purinosome marker (*9, 13*). HeLa cells were cultured in purine-depleted medium (RPMI 1640 medium containing 5% (v/v) dialyzed FBS). Cells were seeded onto 35-mm glass-bottomed dishes (Cellvis) one day prior to transfection. The next day, cells were transfected in OPTI-MEM using FuGENE® 4K reagent (Promega), according to the manufacturer's guidelines. After incubation with FuGENE-DNA complexes for 3 hours, cells were washed with PBS and maintained in fresh purine-depleted medium. Imaging was performed 15–20 hours after transfection at 37°C in a 5% CO_2 environment using a spinning disk confocal system built on a Leica DMI6000 microscope, equipped with a Yokogawa CSU-X1 spinning disk confocal scanner, a Hamamatsu ImagEMX2 EM-CCD camera, and a Leica 100× oil immersion objective lens (NA = 1.49). 20 µM hypoxanthine was added into the medium to induce purinosome disassembly. Purinosome dynamics were monitored by time-lapse imaging at 30-second intervals for 1 hour. Spinning disk images were processed and analyzed using an in-house Fiji/ImageJ macro script. Briefly, raw images were imported into Fiji/ImageJ, and Labkit (*57*) was utilized to segment purinosomes and generate corresponding masks. Purinosome numbers in each image were quantified using the "Analyze Particles" function of Fiji/ImageJ.

To analyze purinosomes using 3D Super-resolution structured illumination microscopy (3D-SIM), cells were fixed using 4% w/v PFA + 0.4% v/v glyoxal + 0.1% v/v methanol in PBS for 10 minutes at room temperature as previously described (*58*), then permeabilized in PBS containing 0.5% Triton X-100 for 5 minutes at room temperature. Cells were blocked with 10% BSA in PBS for 1 hour at room temperature followed by incubation with primary anti-FLAG M2 antibody (F1804, Sigma, RRID: AB_262044,1:200 dilution in 2% BSA in PBS) and NUDT5 antibody (A0609, ABclonal, 1:100 dilution in 2% BSA in PBS) for 1 hour at room temperature. After three washes with PBS, cells were incubated with goat-anti mouse Alexa 568 (A-11031, ThermoFisher) or

goat-anti rabbit (A-21245, ThermoFisher) (1:1,000 dilution in 2% BSA in PBS) for 1 hour at room temperature. Samples were mounted using VECTASHIELD antifade mounting medium (H-1000-10, Vector Lab). Images were collected using a DeltaVision OMX SR system (Cytiva) equipped with a 60x /1.42 NA Plan Apo oil-immersion objective (Olympus) and four laser beams (405, 488, 568 and 642nm; 100mW). The microscope was routinely calibrated with a special image registration slide and algorithm provided by Cytiva. To obtain optimal images, immersion oil with a refractive index of 1.516 was used at 25˚C room temperature. SIM image stacks were captured with a z-distance of 0.125 mm and with 5 phases, 3 angles, and 15 raw images per plane. The raw data were reconstructed with channel specific OTFs and a Wiener filter was set to optimum value by using the softWoRx 6.5 package (Cytiva). Images were registered with alignment parameters obtained from calibration measurements with 100 nm diameter TetraSpeck Microspheres with four colors (Molecular Probes). SIM images were processed and analyzed using an in-house Fiji/ImageJ macro script. Briefly, raw images were imported into Fiji/ImageJ, and the AutoThreshold function was utilized to segment purinosomes with the Yen method and generate corresponding masks. Purinosome volume and PPAT signal were quantified using the "3D Objects Counter" function of Fiji/ImageJ. Colocalization between FGAMS, NUDT5, and PPAT was analyzed using the Coloc module of Imaris, and Pearson's correlation coefficients were calculated to quantify the degree of colocalization.

PPAT oligomerization analysis

Cells were cultured in 10 cm plates until they were more than 90% confluent. Cells were rinsed with 3 mL cold PBS once and scraped into 1 mL cold PBS on ice. Cell pellets were collected by centrifugation at 20,160 x g for 1 min at 4˚C, and supernatants were aspirated. Samples were transferred into a glovebox (30˚C, 0.5% O_2), where all buffers had been degassed overnight. Cell pellets were resuspended in 1mL lysis buffer containing 20 mM HEPES-Na pH 7.5, 150 mM NaCl, 1% Triton-X100 and proteinase and phosphatase inhibitor cocktail and subjected to three cycles of freeze-thaw in liquid nitrogen and water. The tubes were sealed with parafilm, removed from the glovebox for centrifugation at 20,160 x g for 10 minutes at 4˚C. The samples were returned to the glovebox where the supernatants were transferred to new tubes. 10 µL aliquots of each sample were collected for protein quantification. For crosslinking, fresh glutaraldehyde (G5882, Sigma-Aldrich) diluted in the lysis buffer was added to the samples and incubated for 30 minutes in the glovebox. Reactions were quenched with Tris-HCl (pH 7.5, final concentration 100 mM) for 10 minutes in the glovebox, followed by addition of 4x Laemlli buffer without reducing agents (161-0747, Bio-Rad). The mixed samples were immediately loaded onto 4-12% Bis-Tris Protein gels (NP0323BOX, Invitrogen) for immunoblotting. The samples were only boiled at 95˚C for 5 minutes for probing the loading control.

Protein purification

To purify NUDT5 and truncated PPAT (PPAT(1-270)), the cDNAs for full-length human NUDT5, maltose-binding protein, and an N-terminal maltose-binding protein-fused PPAT domain (MBP-PPAT(1 - 270)) were cloned into the pET15b vector containing an N-terminal polyhistidine tag. Site-directly mutagenesis was performed to introduce the R70A mutation into the NUDT5 sequence. NUDT5, MBP and MBP-PPAT plasmids were transformed into *E. coli* BL21 (DE3) (C600003, Invitrogen). The bacteria were grown in

Luria-Bertani (LB) medium at 37˚C, 250-rpm shaking until the optical density at 600 nm (OD_{600}) reached approximately 3.0. Protein expression was induced by 0.4 mM isopropyl ß-D-1-thiogalactopyranoside (IPTG) (I1003, Anatrace) and followed by additional two hours of incubation at 37˚C. For MBP-PPAT(1-270) expression, the bacteria were inoculated and grown until OD_{600}~0.7 before addition of 0.4 mM IPTG at 16˚C for 20 hours. Bacterial pellets were harvested by centrifugation at 4,000 x g at 4˚C for 20 minutes. The pellets were resuspended in lysis buffer containing 20 mM Tris-HCl pH 7.4, 150 mM NaCl, 1% Triton X-100, 0.2 mM PMSF, and protease inhibitors, including 1 µg/ml leupeptin (L2884, Sigma-Aldrich), 1 µg/ml pepstatin (P5318, Sigma-Aldrich), 1 mM benazamidine HCl (12072, Sigma-Aldrich). The resuspended pellets were flash-frozen by liquid nitrogen and stored at -80 ˚C. Frozen bacterial pellets were thawed and subjected to three cycles of freeze-thawing using liquid nitrogen and a 37˚C water bath. Insoluble debris was removed by centrifugation at 20,000 x g for 30 minutes at 4˚C. The target proteins were captured via affinity purification using Ni-NTA agarose on an orbital rotator for 1 hour at 4˚C (30230, Qiagen). The resin was washed with 20 mL of washing buffer containing 20 mM Tris-HCl pH 7.4, 150 mM NaCl, 30 mM imidazole, and eluted with buffer containing 300 mM imidazole pH 7.4. The eluates were purified by size exclusion chromatography equilibrated in a buffer containing 20 mM Tris-HCl pH 7.4, and 150 mM NaCl. To remove the MBP tag from PPAT, 2 mg purified polyhistidine-tagged MBP-PPAT was incubated with 0.1 mg polyhistidine-tagged TEV (Tobacco Etch Virus) protease for 16 hours at room temperature. The TEV protease and the cleaved MBP were then removed by 100 µL 50% Ni-NTA resin. The cleaved PPAT domain was collected from the flow-through, verified by running sodium dodecyl sulfate–polyacrylamide gel electrophoresis (SDS-PAGE) and Coomassie staining. All the purified proteins were verified for high purity (>90%) by running SDS-PAGE and Coomassie staining before being concentrated using molecular-weight cutoff filters: 100-kDa filters (UFC9100, Millipore Sigma) for PPAT and 10-kDa filters (UFC8010D, Millipore Sigma) for NUDT5, MBP and MBP-PPAT. The concentrated proteins were flash-frozen in liquid nitrogen and stored at -80˚C.

To purify full-length human PPAT, human C-terminal Flag/Strep-tagged PPAT cDNA was cloned into a pCDNA5 vector (a gift from Dr. Gerta Hoxhaj's Laboratory at University of Texas, Southwestern Medical center). HEK293F cells were transiently transfected with the PPAT plasmid using polyethyleneimine (PEI) (49553-93-7, Polysciences) following the manufacturer's instructions and cultured at 37˚C, 8% CO_2. After 16 hours, the cells were treated with 100 µM $FeSO_4$ (ferrous sulfate) (F8633, Sigma) and 3 mM sodium butyrate (303410, Sigma). The cells were cultured for additional 56 hours and harvested by centrifugation at 4,000 x g at 4˚C for 20 minutes. The cell pellets were flash-frozen in liquid nitrogen and stored at -80˚C. PPAT purification was performed at room temperature under strict anaerobic conditions in a COY anaerobic chamber (COY Laboratory Products). All buffers used for protein purification were thoroughly degassed under a Schlenk Line and supplemented with 1 mM 1,4-dithiothreitol (DTT) immediately before use. Frozen cell pellets expressing PPAT were thawed and resuspended in lysis buffer containing 20 mM Tris-HCl pH 7.4, 150 mM NaCl, 1 mM DTT, 10 µg/ml lysozyme, 3 µg/ml DNase I, and the cOmplete™ EDTA-free protease inhibitor cocktail (Millipore Sigma) per instructions by the manufacturer. The cells were lysed by sonication, and insoluble cellular debris was removed by centrifugation at 20,000 x g for 45 minutes at

4˚C. The clarified lysate was incubated with 2 ml 50% slurry anti-Flag M2 resins (A2220, Sigma-Aldrich) for 1 hour, at 4˚C on an orbital rotator to capture the Flag-tagged PPAT protein. The resin was washed three times with 20 ml of a washing buffer containing 20 mM Tris-HCl pH 7.4, 150 mM NaCl, and 1 mM DTT. The bound protein was eluted by an elution buffer containing 20 mM Tris-HCl pH 7.4, 150 mM NaCl, 1 mM DTT, and 200 μg/ml Flag peptide (A6001, ApexBio).

In vitro binding assay

100 μg of MBP-PPAT(1-270) or MBP protein was immobilized on 50 μl of 50% slurry amylose resin (E8021l, NEB) by 30 minutes of incubation at room temperature. The resin was washed three times with TBST buffer (20 mM Tris-HCl pH 7.5, 150 mM NaCl, 0.005% Tween-20) to remove any unbound protein. 100 μg of NUDT5 was added to the MBP-PPAT(1-270)- or MBP-bound resin and incubated for 30 minutes at room temperature. After five washes with TBST, bound proteins were eluted by 50 μl of TBST containing 10 mM maltose (BP684-500, Fisher Scientific) for 15 minutes at room temperature. To confirm binding of the cleaved PPAT domain to NUDT5, a reversed pull-down was conducted. 20 μg of NUDT5 protein was incubated with 50 μl of 50% slurry Ni-NTA resins (30230, Qiagen) for 30 minutes at room temperature. After three washes with TBST, 20 μg of cleaved PPAT was added and incubated for 30 minutes at room temperature. Following five washes with TBST, the bound proteins were eluted with 50 μl of TBST containing 300 mM imidazole pH 8.0 for 15 minutes at room temperature. A negative control was performed using Ni-NTA resin without NUDT5 protein. All the eluates were analyzed by immunoblotting.

PPAT enzymatic activity assay

For the cell lysate-based assay, confluent cells from one 10 cm plate were harvested, and lysates were prepared as described in **PPAT oligomerization analysis**. Lysates were diluted in 1× TBST and 200 μg protein was used per reaction. For each cell line, two 100 μL reactions were set up in the glovebox (30˚C, 0.5% O_2): one containing 200 μM [U-^{13}C]glutamine, 200 μM PRPP, and 10 mM $MgCl_2$, and the other containing all components except PRPP. After 3 hours of incubation, the reactions were quenched with EDTA (pH 8.0, final concentration 40 mM). Samples were removed from the glovebox, mixed with 400 μL 100% LC-MS–grade methanol containing 250 ng/mL [2,3,3-$^2H_3$]aspartate (internal standard) (DLM-832-PK, Cambridge Isotope Laboratories), and centrifuged at 20,160 × g for 15 minutes at 4˚C. Supernatants were dried in a SpeedVac overnight, resuspended in 100 μL 80% acetonitrile, and filtered through a Waters Oasis HLB 96-well plate (50-818-654, Fisher Scientific) pre-washed with water and 80% acetonitrile. Residuals were further washed with 500 μL 80% acetonitrile, and eluates were centrifuged at 20,160 × g for 15 min at 4˚C. Final supernatants were analyzed by LC-MS using an AB SCIEX QTRAP 5500 triple quadrupole system (Applied Biosystems SCIEX) as previously described (*59*). The abundance of [U-^{13}C]glutamate was normalized to the internal standard, and PPAT activity was calculated as the ratio of [U-^{13}C]glutamate between reactions with and without PRPP.

For the assay using purified proteins, a reaction mixture containing 50 nM PPAT, 200 μM PRPP, 200 μM glutamine, and 10 mM $MgCl_2$ in TBST was incubated for 3 hours in a glovebox (30˚C, 0.5% O_2). To investigate the effect of NUDT5, AMP, and GMP on PPAT activity, 100 nM NUDT5, 25 μM AMP, or 25 μM GMP were added to the reaction mixture. Control reactions were performed under identical conditions without PPAT. After

terminating the reaction by EDTA (pH 8.0, final concentration 40 mM), the samples were removed from the glovebox and glutamate was quantified using a fluorometric assay (STA-674, Cell Biolabs) as previously described (*60*).

Statistical analysis

Data from samples with cross-contamination during preparation were excluded. The exclusion of data points was not due to attribution or intentional exclusion. Figures were prepared and statistics were calculated using GraphPad PRISM and R. Statistical calculation details are indicated in the figure legends for each figure. *P < 0.05; **P < 0.01; ***P < 0.001; ****P < 0.0001; n.s.; not significant (P > 0.05).

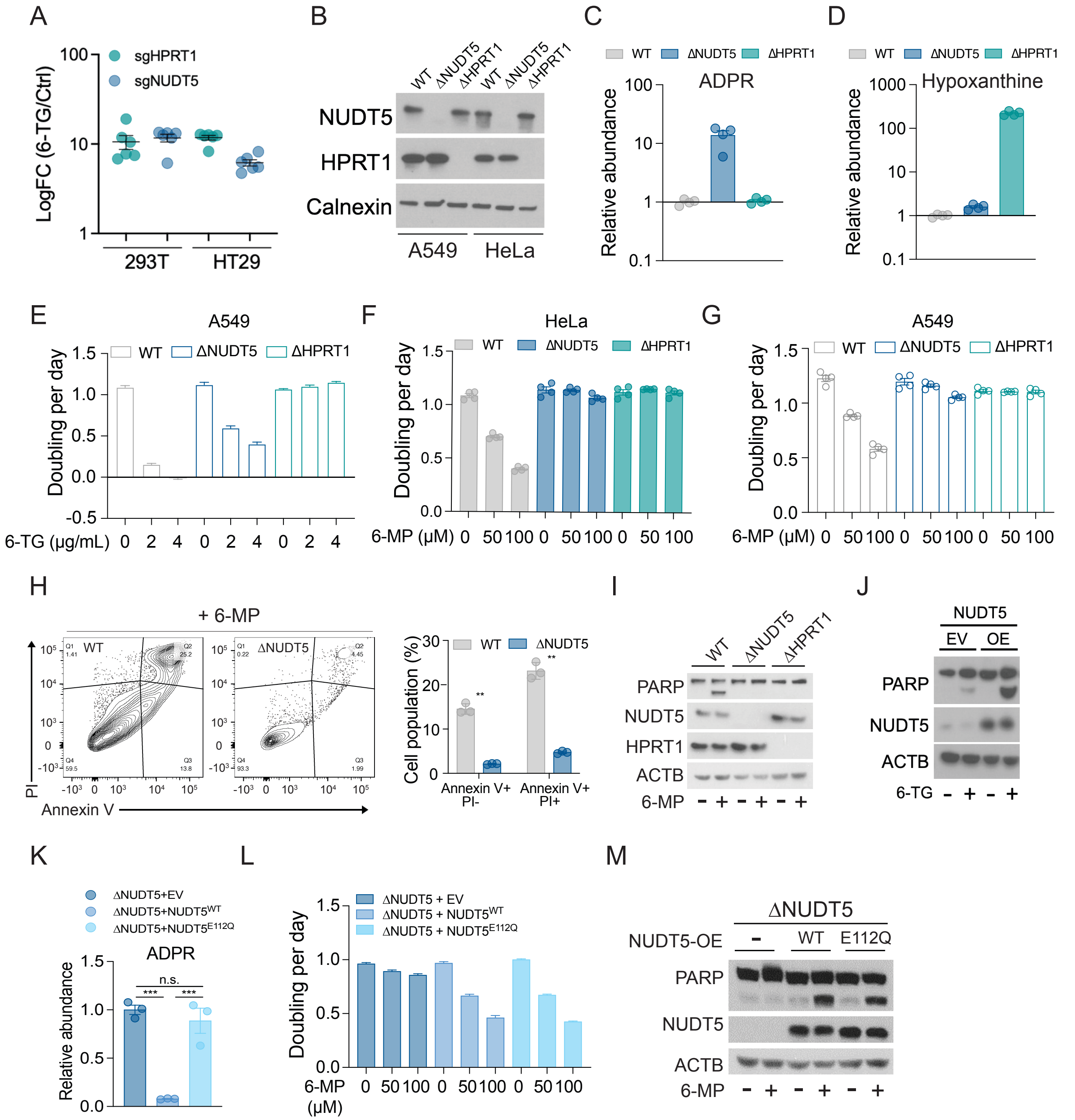

Figure S1. NUDT5 regulates cellular sensitivity to thiopurines independently of its catalytic function. (A) Enrichment of the indicated gRNA in 6-TG-treated cells during genome-wide CRISPR screen. Data are from Doech et al. (*15*). **(B)** Western blot validating depletion of HPRT1 and NUDT5 in HeLa and A549 cells. Calnexin is the loading control. **(C-D)** Relative abundance of ADPR (C) and hypoxanthine (D) in WT, ΔNUDT5, and ΔHPRT1 HeLa cells (n=4). **(E)** Growth rates of WT, ΔNUDT5, and ΔHPRT1 A549 cells treated with indicated doses of 6-TG. Data are technical replicates from one of three

independent experiments. **(F-G)** Growth rates of WT, ∆NUDT5, and ∆HPRT1 HeLa (F) and A549 (G) cells treated with the indicated doses of 6-MP. Data are technical replicates from one of three independent experiments. **(H)** Apoptosis analysis in WT and ΔNUDT5 HeLa cells treated with 20 μM 6-MP for 48 hours. To the left are representative contour plots. To the right are bar graphs showing the percentage of apoptotic (Annexin V+, PI-) and dead (Annexin V+, PI+) cells. (n=3). **(I)** Western blot assessing cleaved PARP in WT, ΔNUDT5, and ΔHPRT1 HeLa cells treated with or without 20 μM 6-MP for 24 hours. β-actin (ACTB) is the loading control. **(J)** Western blot assessing cleaved PARP in WT HeLa cells expressing an empty vector (EV) or overexpressing (OE) NUDT5. Cells were treated with or without 0.5 μg/mL 6-TG for 24 hours. β-actin (ACTB) is the loading control. **(K)** Relative abundance of ADPR in ∆NUDT5 HeLa cells that express empty vector (EV), $NUDT5^{WT}$, or $NUDT5^{E112Q}$. (n=3). **(L)** Growth rates of ∆NUDT5 HeLa cells that express empty vector (EV), $NUDT5^{WT}$, or $NUDT5^{E112Q}$ treated with the indicated doses of 6-MP. Data are technical replicates from one of three independent experiments. **(M)** Western blot assessing cleaved PARP in ∆NUDT5 HeLa cells that express empty vector (EV), $NUDT5^{WT}$, or $NUDT5^{E112Q}$ treated with 20 μM 6-MP for 24 hours. β-actin (ACTB) is the loading control. Data points in each panel represent an independent sample unless specified. Error bars denote SEM. Multiple t test (H) and One-way ANOVA (K) were used for statistical analysis. ***: $P < 0.001$; n.s.: $P > 0.05$.

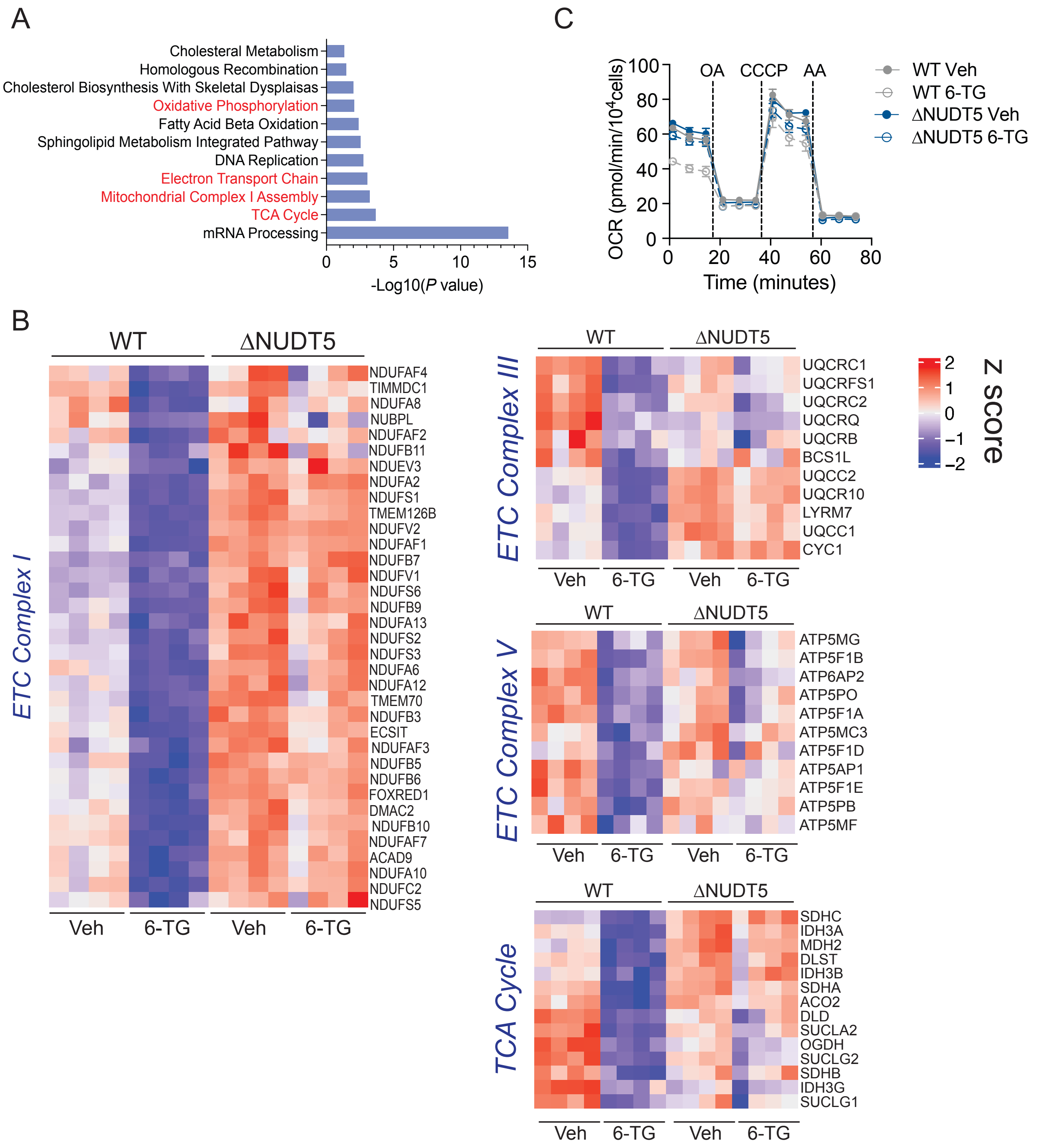
A
Cholesteral Metabolism
Homologous Recombination
Cholesterol Biosynthesis With Skeletal Dysplaisas
Oxidative Phosphorylation
Fatty Acid Beta Oxidation
Sphingolipid Metabolism Integrated Pathway
DNA Replication
Electron Transport Chain
Mitochondrial Complex I Assembly
TCA Cycle
mRNA Processing
-Log10(P value)
C
OA
CCCP
AA
WT Veh
WT 6-TG
ΔNUDT5 Veh
ΔNUDT5 6-TG
OCR (pmol/min/10⁴cells)
Time (minutes)
B
WT
ΔNUDT5
ETC Complex I
Veh
6-TG
ETC Complex III
ETC Complex V
TCA Cycle
Z score

Figure S2. 6-TG leads to depleted mitochondrial ETC subunits and reduced respiration in a NUDT5-dependent manner. (A) Proteomics analysis showing down-regulated pathways in 6-TG-treated WT cells compared to vehicle-treated WT, vehicle-treated ∆NUDT5 and 6-TG-treated ∆NUDT5 cells. **(B)** Heatmaps showing abundance of proteins in ETC complexes and the TCA cycle in WT and ∆NUDT5 HeLa cells treated with or without 0.5 µg/mL 6-TG for 24 hours. (n=4) **(C)** Oxygen consumption rates of HeLa cells pre-treated with vehicle or 0.5 µg/mL 6-TG. OA: oligomycin A; CCCP: carbonyl cyanide m-chlorophenylhydrazone; AA: antimycin A. Data are technical replicates from one of three independent experiments. Data points in each panel represent an independent sample unless specified. Error bars denote SEM.

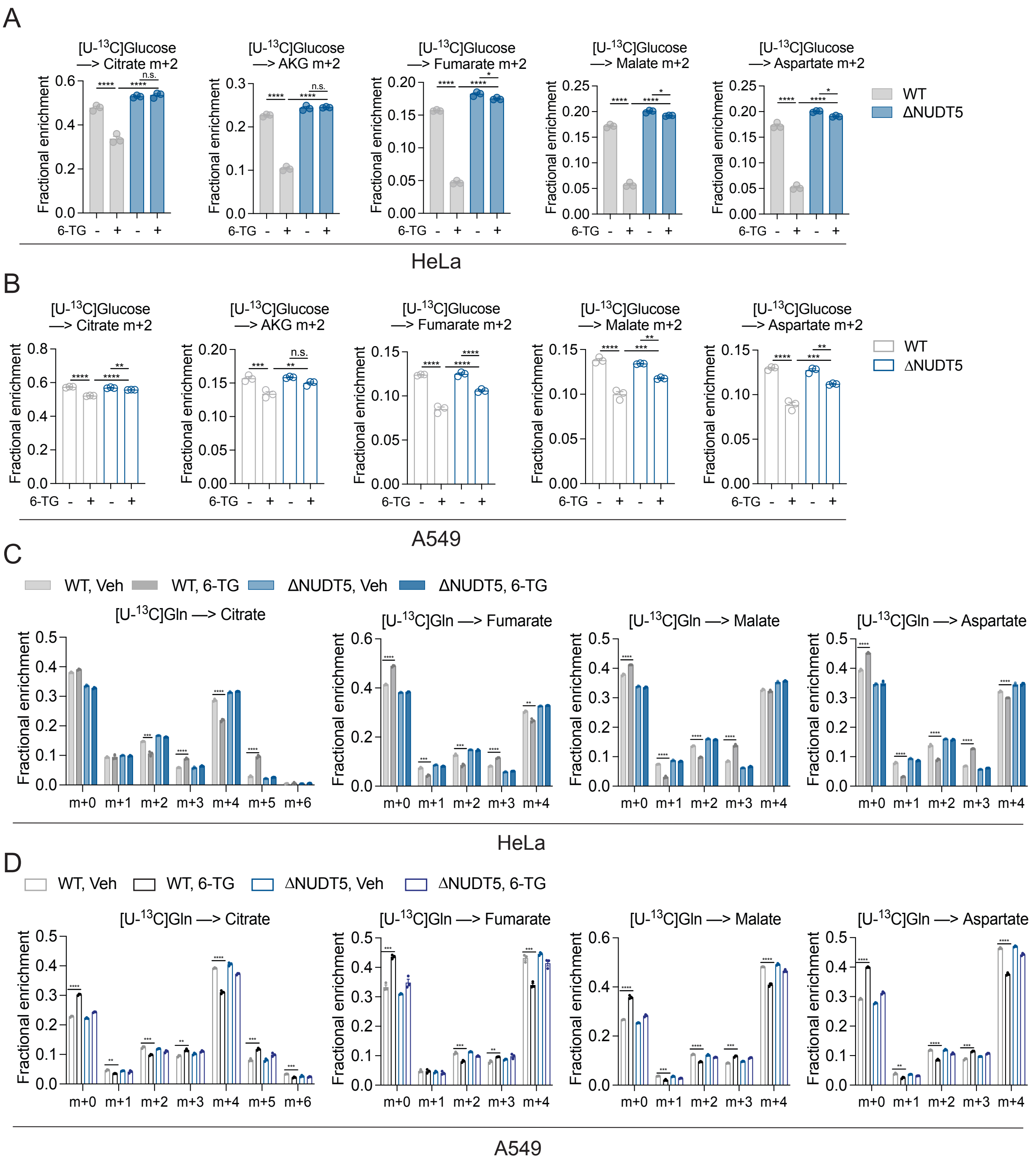

Figure S3. 6-TG alters glucose and glutamine metabolism in a NUDT5-dependent fashion. (A-B) Isotopologue fractions in citrate, α-ketoglutarate (AKG), fumarate, malate, and aspartate after 6 hours of culture with [U-^{13}C]glucose in WT and ΔNUDT5 HeLa (A) and A549 (B) cells pre-treated with vehicle or 0.5 μg/mL 6-TG for 24 hours. (n=3). **(C-D)** Isotopologue fractions in citrate, fumarate, malate, and aspartate after 6 hours of culture with [U-^{13}C]glutamine in WT and ΔNUDT5 HeLa (C) or A549 (D) cells pre-treated with vehicle or 0.5 μg/mL 6-TG for 24 hours. (n=3). Data points in each panel represent an

independent sample unless specified. Error bars denote SEM. One-way ANOVA (A-B) and multiple unpaired two-sided t tests (C-D) were used for the statistical analysis. ****: $P < 0.0001$; ***: $P < 0.001$; **: $P < 0.01$.

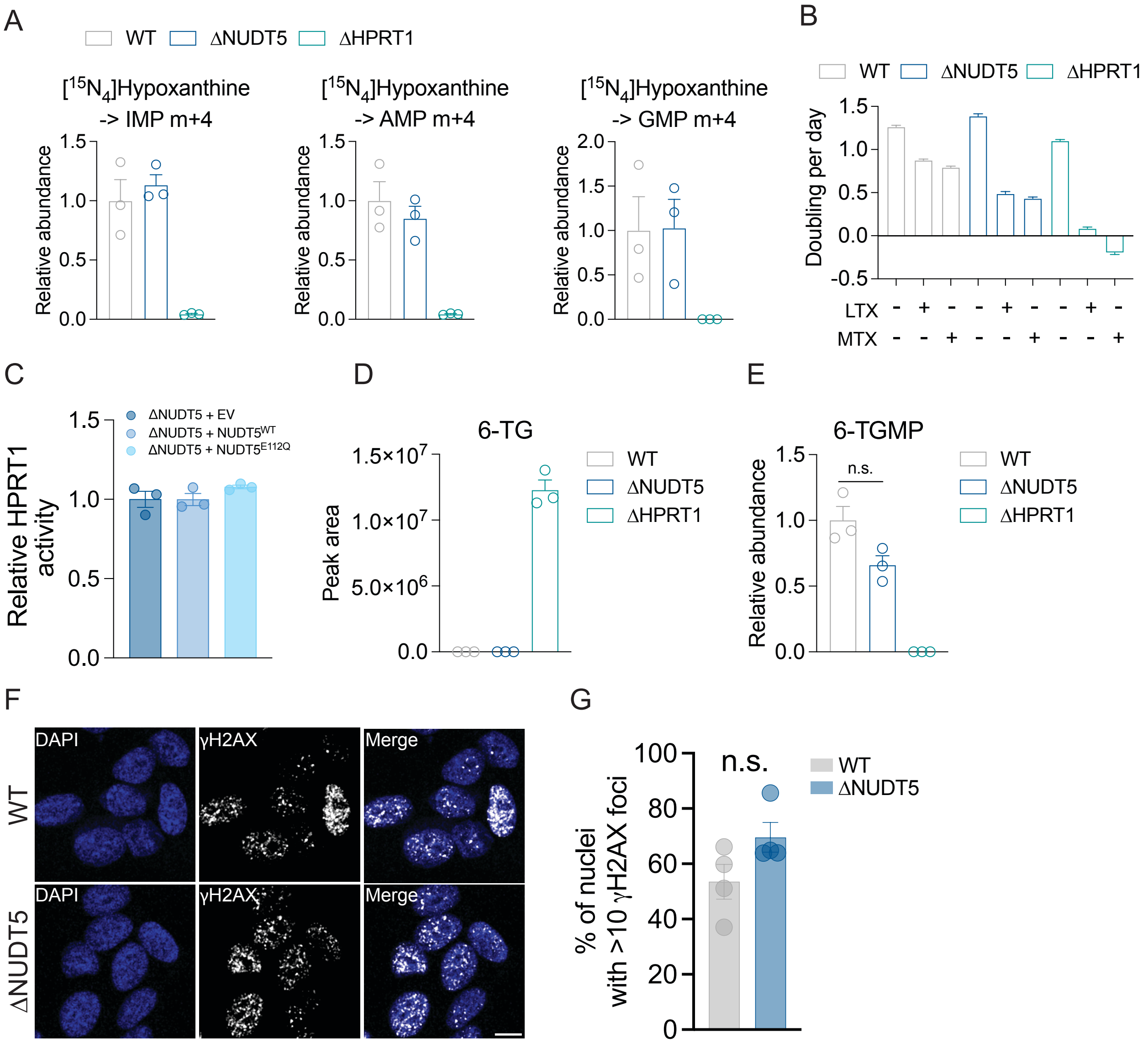

Figure S4. NUDT5 is not required for purine salvage and 6-TG metabolism. (A) Relative abundance of m+4 IMP, m+4 AMP, and m+4 GMP from [$^{15}N_4$]hypoxanthine in WT, ΔNUDT5, and ΔHPRT1 A549 cells during 4 hours of tracing. (n=3). **(B)** Growth rates of WT, ΔNUDT5, and ΔHPRT1 A549 cells treated with DMSO, 1 μM LTX, or 1 μM MTX. Data are technical replicates from one of three independent experiments. **(C)** Relative HPRT1 enzymatic activity in lysates from ΔNUDT5 HeLa cells that express empty vector (EV), NUDT5^{WT}, or NUDT5^{E112Q}. (n=3). **(D)** Intracellular 6-TG abundance (peak area) in WT, ΔNUDT5, and ΔHPRT1 A549 cells treated with 0.5 μg/mL 6-TG for 24 hours. No peaks were detected in the WT and ΔNUDT5 cells. (n=3). **(E)** Relative abundance of 6-TGMP in WT, ΔNUDT5, and ΔHPRT1 A549 cells treated with 0.5 μg/mL 6-TG for 24 hours. (n=3). **(F)** Analysis of γH2AX foci (white) in WT and ΔNUDT5 HeLa cells treated with 0.5 μg/mL 6-TG for 48 hours. Nuclei were labeled with DAPI (blue). The scale bar represents 10 μm. **(G)** Quantification of nuclei containing more than 10 γH2AX foci in (F).

(n=4 independent experiments). Data points in each panel represent an independent sample unless specified. Error bars denote SEM. Unpaired two-sided t tests were used for statistical analysis (E and G). n.s.: $P > 0.05$.

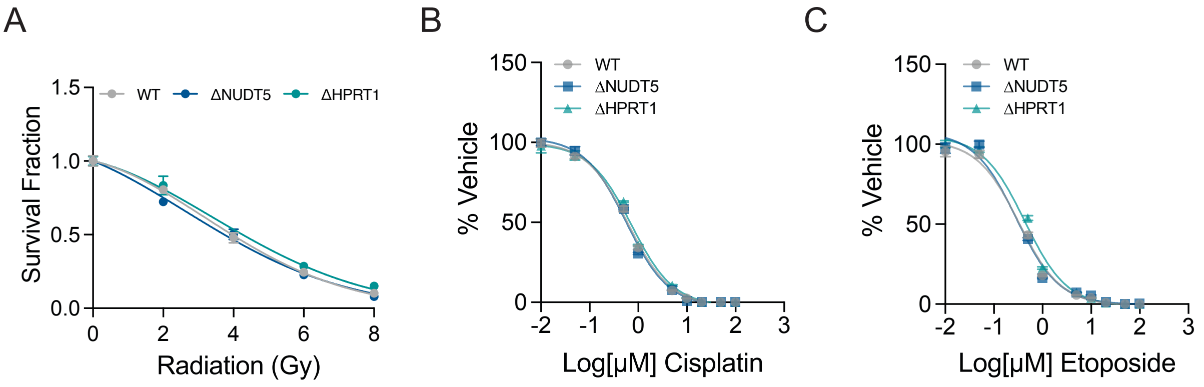

Figure S5. NUDT5 loss does not confer resistance to ionizing radiation or general DNA damaging agents. (A) Relative survival fraction of WT, ΔNUDT5, and ΔHPRT1 HeLa cells treated with the indicated doses of ionizing radiation (IR). Data are normalized to the untreated group. (n=3). **(B-C)** Relative survival of WT, ΔNUDT5, and ΔHPRT1 HeLa cells treated with the indicated doses of cisplatin (B) or etoposide (C). Data are normalized to the vehicle-treated groups. Data are technical replicates from one of three experiments. Error bars denote SEM.

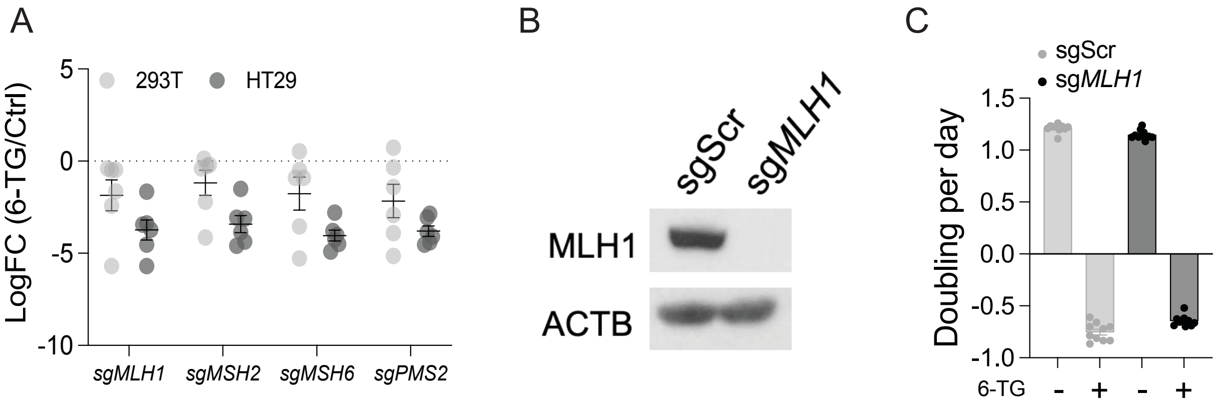

Figure S6. Suppressing the DNA mismatch repair (MMR) pathway does not confer thiopurine resistance. (A) Enrichment of the indicated gRNAs against MMR genes in 6-TG-treated cells during genome-wide CRISPR screen. Data are from Doench et al. (*15*). **(B)** Western blot validating depletion of MLH1. β-actin (ACTB) is the loading control. **(C)** Growth rates of HeLa cells expressing a scrambled gRNA (sgScr) or a gRNA against *MLH1* (sg*MLH1*) treated with vehicle or 4 μg/mL 6-TG. Data are technical replicates from one of three experiments. Error bars denote SEM.

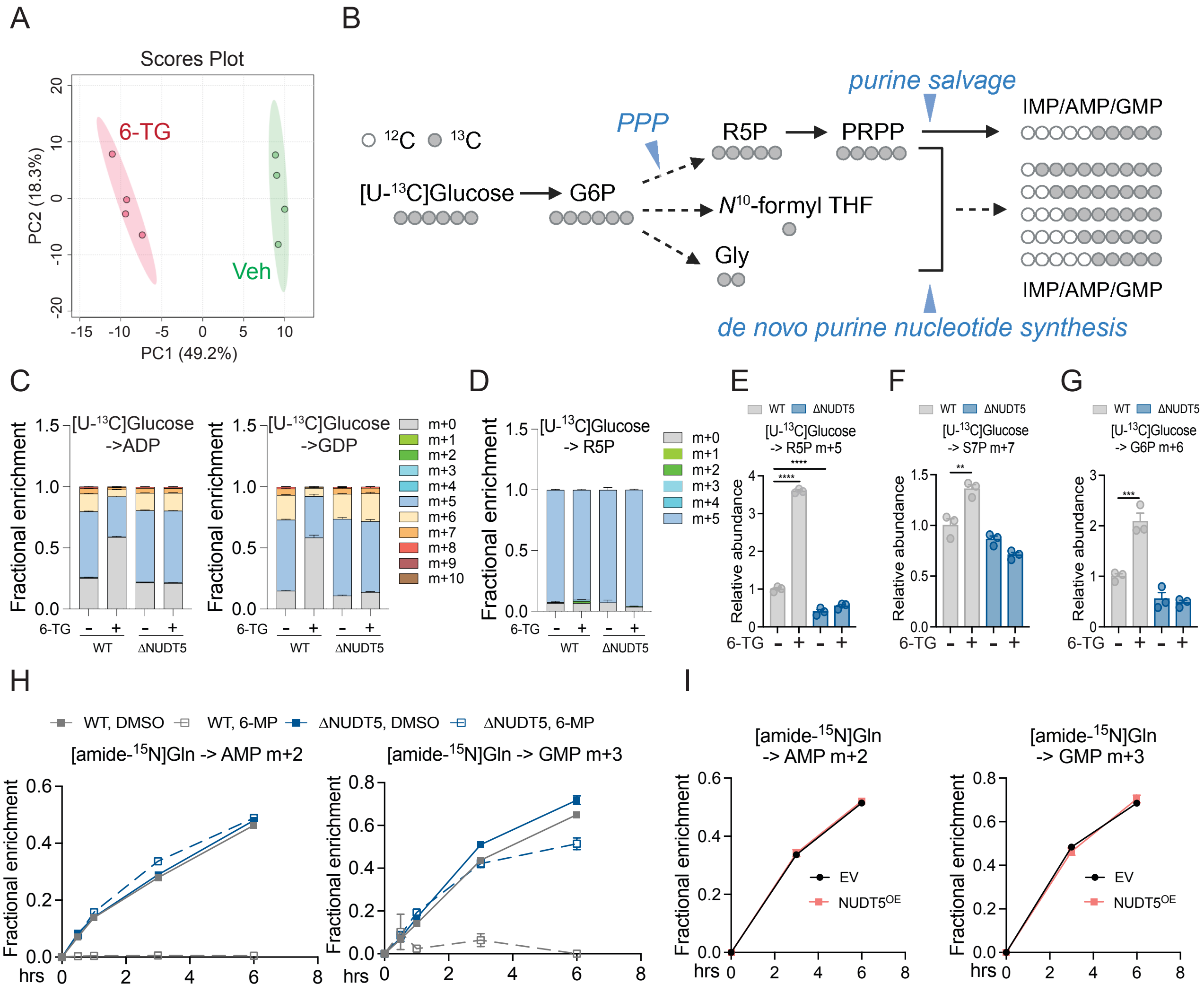

Figure S7. Metabolic effects of 6-TG. (A) Principal component analysis of metabolomic profiles in WT HeLa cells treated with vehicle (Veh) or 0.5 µg/mL 6-TG for 24 hours. **(B)** Schematic illustrating labeling of purine nucleotides from [U-^{13}C]glucose. **(C)**^{13}C labeling in the indicated purine nucleotides after 6 hours of culture with [U-^{13}C]glucose in WT and ΔNUDT5 HeLa cells pre-treated with vehicle or 0.5 µg/mL 6-TG for 24 hours. (n=3). **(D-G)** ^{13}C labeling in ribose 5-phosphate (R5P) (D) and relative abundance of m+5 R5P (E), m+7 sedoheptulose 7-phosphate (S7P) (F), and m+6 glucose 6-phosphate (G6P) (G) from [U-^{13}C]glucose during 6 hours of tracing in WT and ΔNUDT5 HeLa cells pre-treated with or without 0.5 µg/mL 6-TG for 24 hours. (n=3). **(H)** Time-dependent fractional enrichment of m+2 AMP and m+3 GMP from [amide-^{15}N]glutamine in WT and ΔNUDT5 HeLa cells pre-treated with 20 µM 6-MP for 24 hours. (n=3). **(I)** Time-dependent fractional enrichment of m+2 AMP and m+3 GMP from [amide-^{15}N]glutamine in WT HeLa cells that overexpress empty vector (EV) or NUDT5. (n=3). Data points in each panel represent an independent sample unless specified. Error bars denote SEM. Unpaired two-sided t test was used for the statistical analysis (E-G). ****: P < 0.0001; ***: P < 0.001; **: P < 0.01.

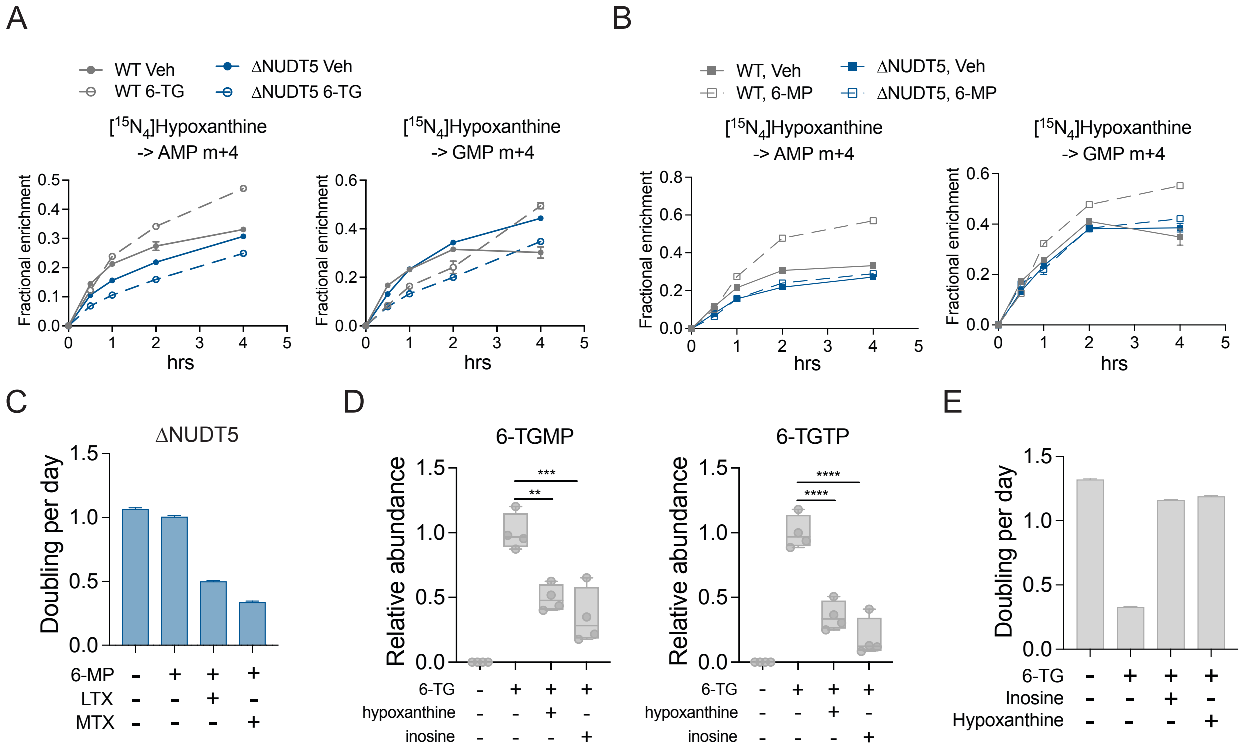

Figure S8. Sustaining purine nucleotide supply is critical for cells to survive thiopurine treatment. (A-B) Time-dependent fractional enrichment of m+4 AMP and m+4 GMP from [$^{15}N_4$]hypoxanthine in WT and ΔNUDT5 HeLa cells pre-treated with vehicle, 0.5 µg/mL 6-TG (A) or 20 µM 6-MP (B) for 24 hours. (n=3). **(C)** Growth rates of ΔNUDT5 HeLa cells treated with or without 100 µM 6-MP, alone or in combination with 1 µM LTX or 1 µM MTX. Data are technical replicates from one of three experiments. **(D)** Relative abundance of 6-TGMP and 6-TGTP in WT HeLa cells treated with or without 0.5 µg/mL 6-TG, alone or in combination with 50 µM hypoxanthine or 50 µM inosine for 24 hours. (n=4). **(E)** Growth rates of WT HeLa cells treated with or without 0.5 µg/mL 6-TG, alone or in combination with 50 µM inosine or 50 µM hypoxanthine. Data are technical replicates from one of three experiments. Data points in each panel represent an independent sample unless specified. Error bars denote SEM. One-way ANOVA was used for the statistical analysis (D). ****: $P < 0.0001$; ***: $P < 0.001$; **: $P < 0.01$.

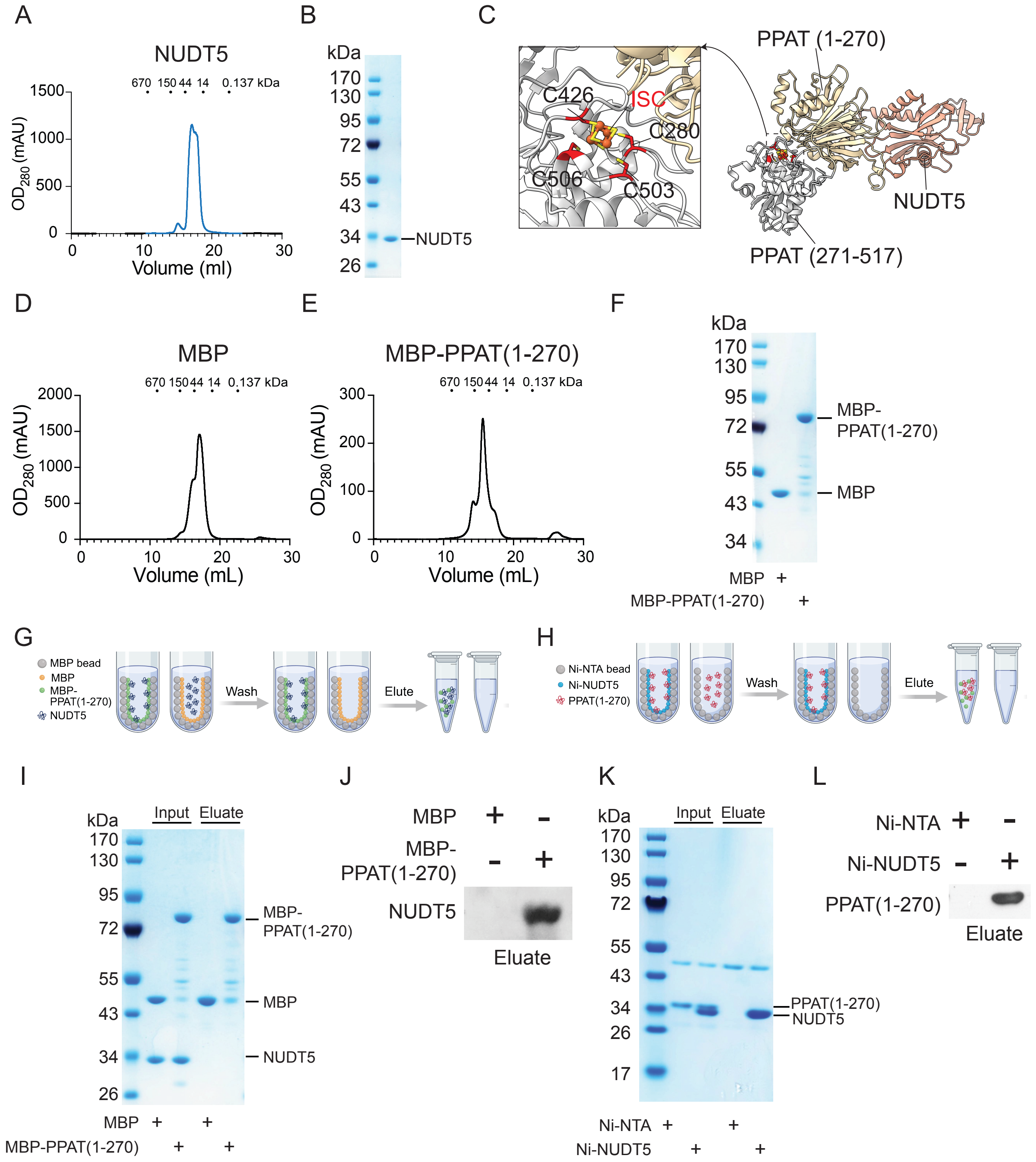

Figure S9. NUDT5 directly binds PPAT. (A) A chromatogram showing purified NUDT5. **(B)** A coomassie blue-stained SDS-PAGE gel showing purified NUDT5. **(C)** Iron-sulfur cluster (ISC) binding domain and NUDT5 binding domain of PPAT predicted by AlphaFold3. **(D-E)** Chromatograms showing purified MBP (D) and MBP-PPAT (1-270) (E). **(F)** A coomassie blue-stained SDS-PAGE gel showing purified MBP and MBP-PPAT (1-270). **(G-H)** Schematics illustrating in vitro binding assays using purified MBP-PPAT

(1-270) (G) or purified NUDT5 (H). **(I)** A coomassie blue-stained SDS-PAGE gel showing MBP-PPAT (1-270), MBP, and NUDT5 in the protein mixtures and eluates. **(J)** Western blot detecting NUDT5 in the MBP-PPAT (1-270) eluate. **(K)** A coomassie blue-stained SDS-PAGE gel showing NUDT5 and PPAT(1-270) in the protein mixtures and Ni-NUDT5 eluates. **(L)** Western blot detecting PPAT (1-270) in the Ni-NUDT5 eluate. BioRender was used to generate the illustration in (G and H).

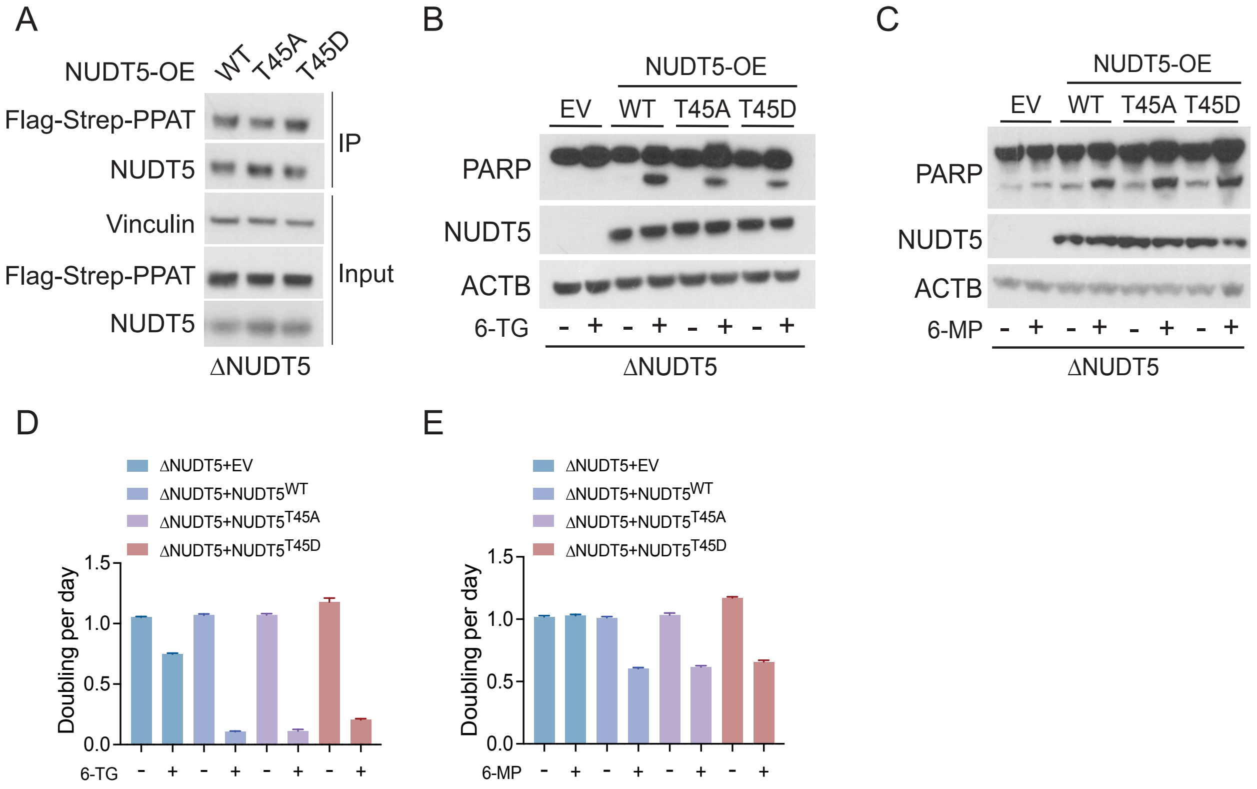

Figure S10. Mutating T45 on NUDT5 does not affect PPAT-NUDT5 interaction or thiopurine sensitivity. (A) Western blot showing the interaction between Flag-Strep-PPAT and NUDT5 in ΔNUDT5 cells expressing $NUDT5^{WT}$, $NUDT5^{T45A}$, and $NUDT5^{T45D}$. Vinculin is the loading control for the input samples. **(B-C)** Western blot assessing cleaved PARP in ΔNUDT5 HeLa cells that express empty vector (EV), $NUDT5^{WT}$, $NUDT5^{T45A}$, or $NUDT5^{T45D}$, treated with 0.5 μg/mL 6-TG (B) or 20 μM 6-MP (C) for 24 hours. β-actin (ACTB) is the loading control. **(D-E)** Growth rates of ΔNUDT5 HeLa cells that express empty vector (EV), $NUDT5^{WT}$, $NUDT5^{T45A}$, or $NUDT5^{T45D}$, treated with 2 μg/mL 6-TG (D) or 100 μM 6-MP (E). Data are technical replicates from three experiments.

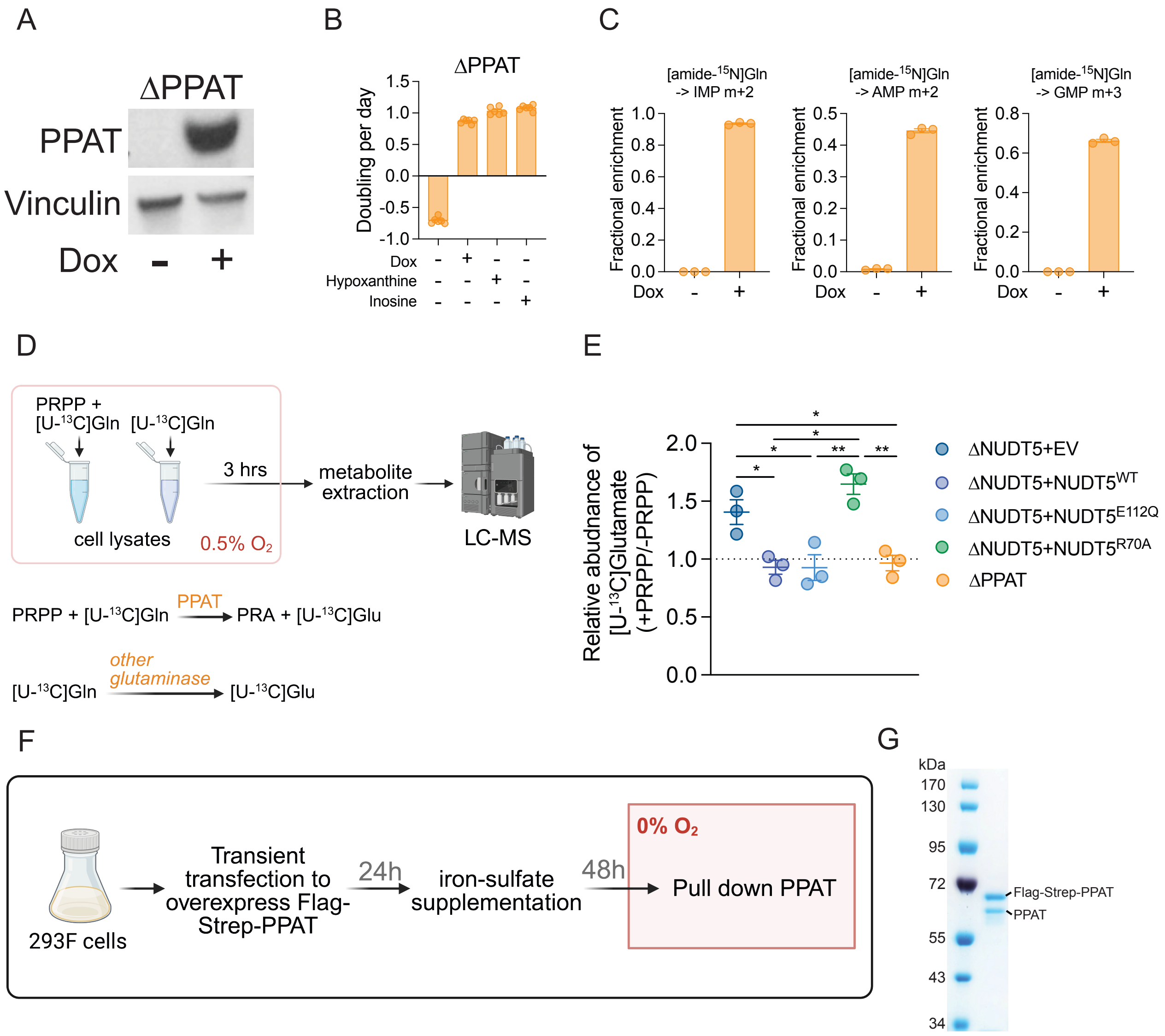

Figure S11. PPAT enzymatic activity analysis in cell lysates. (A) Western blot validating PPAT expression under control of 100 ng/mL doxycycline (Dox) in ΔPPAT HeLa cells. Vinculin is the loading control. **(B)** Growth rates of ΔPPAT HeLa cells cultured in dialyzed FBS (dFBS)-supplemented medium in the presence or absence of 100 ng/mL Dox, 50μM hypoxanthine, or 50 μM inosine. Data are technical replicates from three experiments. **(C)** Fractional enrichment of m+2 IMP, m+2 AMP, and m+3 GMP from [amide-^{15}N]glutamine after 4 hours of incubation in ΔPPAT HeLa cells cultured in the presence or absence of Dox. (n=3). **(D)** Schematic illustrating PPAT enzymatic activity analysis in cell lysates. PRPP, phosphoribosyl-1-pyrophosphate; PRA, 5-phosphoribosyl-1-amine. **(E)** Relative abundance of m+5 glutamate from PRPP-supplemented cell lysates. (n=3). **(F)** Schematic illustrating purification of human full-length PPAT from HEK293F cells. **(G)** A coomassie blue-stained SDS-PAGE gel showing purified human full-length PPAT. Data points in each panel represent an independent sample unless

specified. Error bars denote SEM. One-way ANOVA test was used for the statistical analysis (E). **: P < 0.01; *: P < 0.05. BioRender was used to generate the illustration in (D and F).

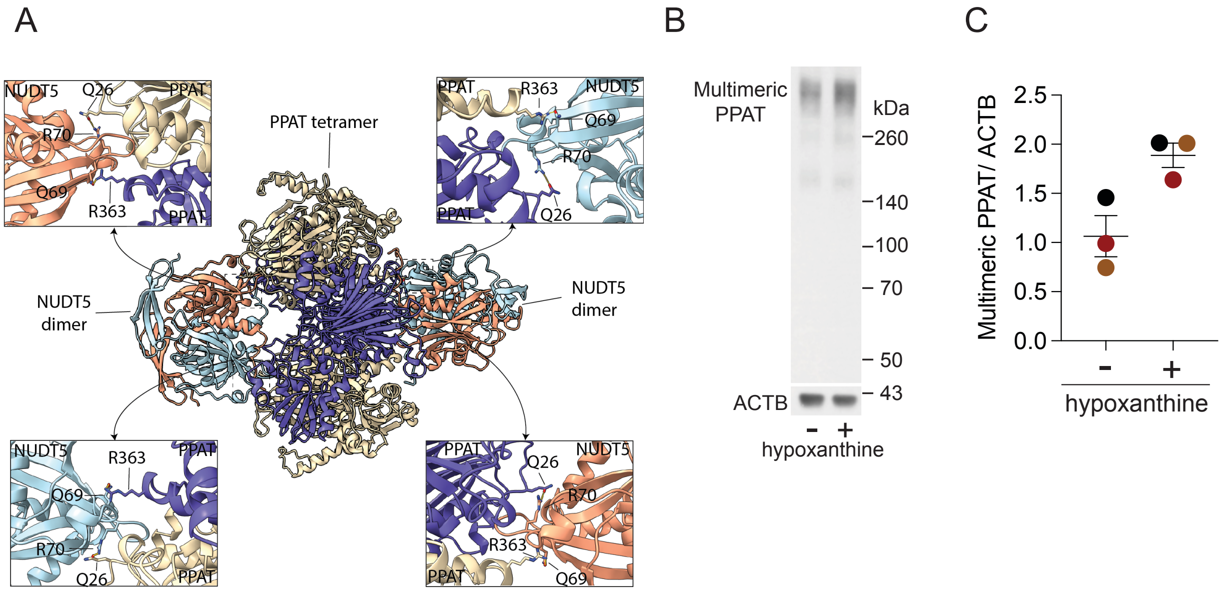

Figure S12. Hypoxanthine repletion modestly enhances PPAT oligomerization. (A) Interactions between NUDT5 dimers and PPAT tetramers depicted by AlphaFold3. **(B)** Western blot showing oligomerization of PPAT in WT HeLa cells cultured in dFBS-supplemented medium and treated with or without 20 μM hypoxanthine for 1 hour. β-actin (ACTB) is the loading control. **(C)** Quantification of mutimeric PPAT abundance in cells described in (B). The multimeric PPAT intensity was normalized to ACTB intensity. Dots with the same color are from the same biological replicate. (n=3).

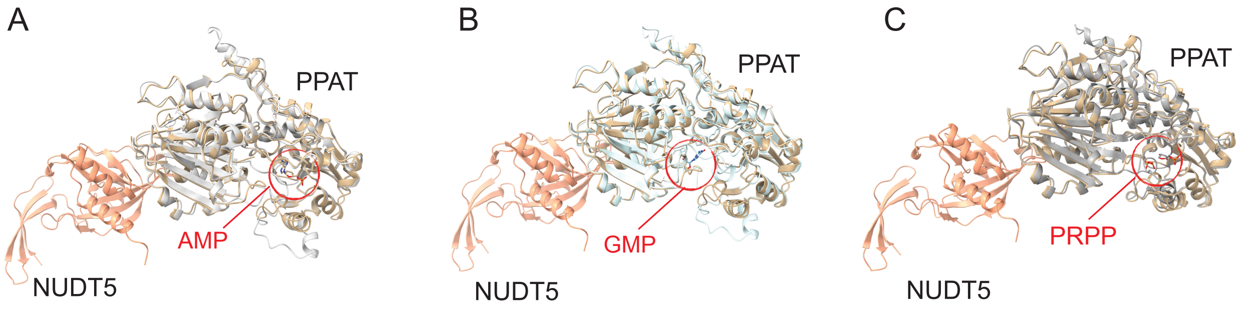

Figure S13. The NUDT5-PPAT interaction is remote from PPAT binding pockets for PRPP and purine nucleotides. (A-C) Superposition of the AlphaFold-predicted PPAT structure in its AMP- (A), GMP- (B), and PRPP- (C) bound forms, complexed with NUDT5.

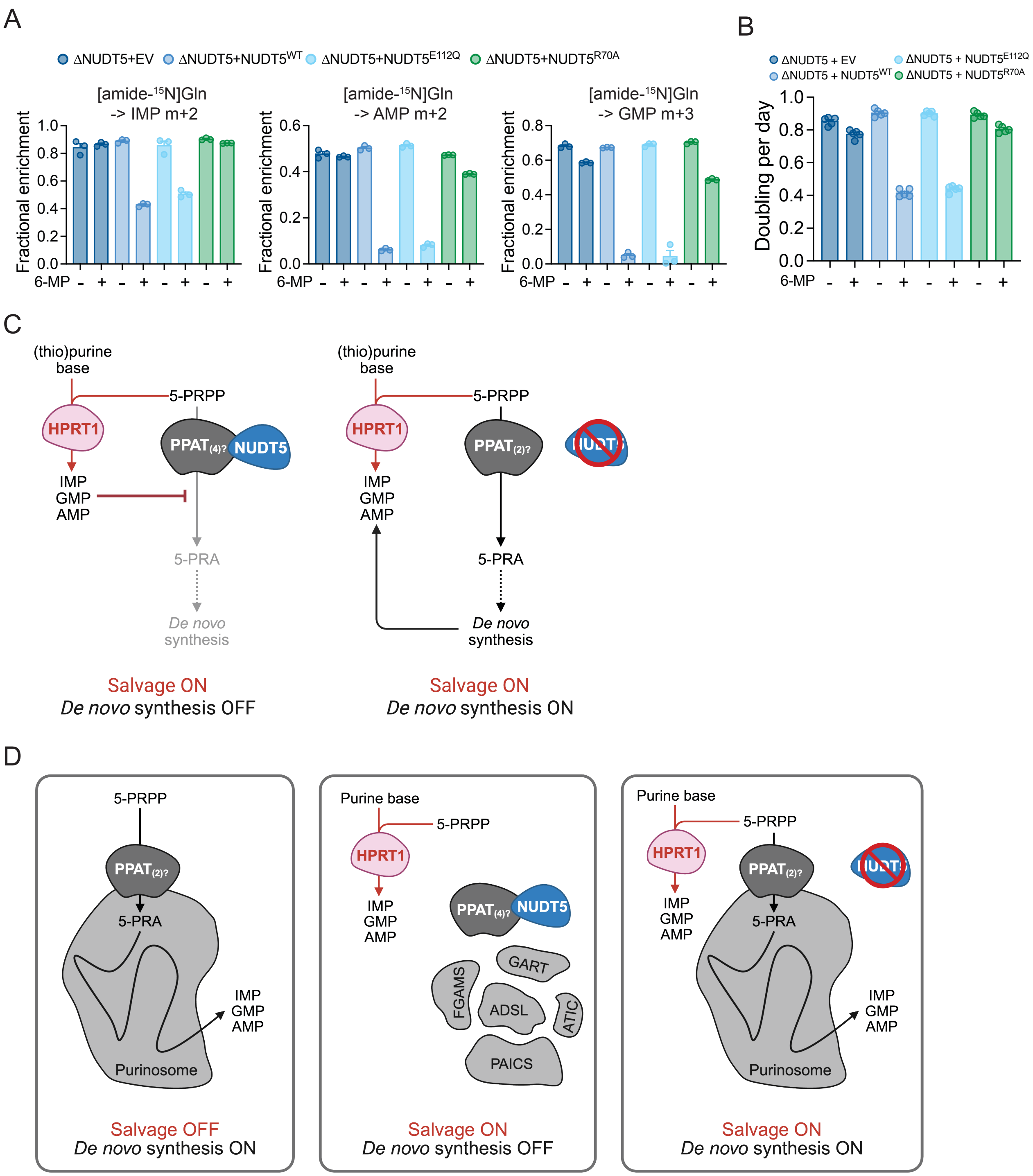

Figure S14. Disruption of the NUDT5-PPAT interaction confers thiopurine resistance. (A) Fractional enrichment of m+2 IMP, m+2 AMP, and m+3 GMP from [amide-^{15}N]glutamine in ΔNUDT5 HeLa cells expressing empty vector (EV), NUDT5^{WT}, NUDT5^{E112Q}, or NUDT5^{R70A} during 6 hours of tracing. The cells were pre-treated with or without 20 μM 6-MP for 24 hours. (n=3). **(B)** Growth rates of ΔNUDT5 HeLa cells that express empty vector (EV), NUDT5^{WT}, NUDT5^{E112Q}, or NUDT5^{R70A} treated with or without

100 µM 6-MP. Data are technical replicates from one of three experiments. **(C-D)** Models of NUDT5 regulating PPAT enzymatic activity (C) and purinosome dynamics (D) during purine salvage. GART: phosphoribosylglycinamide formyltransferase, phosphoribosylglycinamide synthetase, phosphoribosylaminoimidazole synthetase; PAICS: Phosphoribosylaminoimidazole Carboxylase and Phosphoribosylaminoimidazolesuccinocarboxamide Synthase; ADSL: Adenylosuccinate Lyase. ATIC: 5-Aminoimidazole-4-Carboxamide Ribonucleotide Formyltransferase/IMP Cyclohydrolase. FGAMS: phosphoribosylformylglycinamidine synthase. Data points in each panel represent an independent sample unless specified. Error bars denote SEM. BioRender was used to generate the illustration in (C and D).

Movie 1: Imaging of GFP-FGAMS (green) in WT HeLa cells cultured in dFBS-supplemented medium upon 20 μM hypoxanthine treatment. Images were captured at 30-second intervals for 1 hour.

Movie 2: Imaging of GFP-FGAMS (green) in ΔHPRT1 HeLa cells cultured in dFBS-supplemented medium upon 20 μM hypoxanthine treatment. Images were captured at 30-second intervals for 1 hour.

Movie 3: Imaging of GFP-FGAMS (green) in ΔNUDT5 HeLa cells cultured in dFBS-supplemented medium upon 20 μM hypoxanthine treatment. Images were captured at 30-second intervals for 1 hour.

Movie 4: Imaging of GFP-FGAMS (green) in ΔNUDT5 HeLa cells that express an empty vector, cultured in dFBS-supplemented medium upon 20 μM hypoxanthine treatment. Images were captured at 30-second intervals for 1 hour.

Movie 5: Imaging of GFP-FGAMS (green) in ΔNUDT5 HeLa cells that express $NUDT5^{WT}$, cultured in dFBS-supplemented medium upon 20 μM hypoxanthine treatment. Images were captured at 30-second intervals for 1 hour.

Movie 6: Imaging of GFP-FGAMS (green) in ΔNUDT5 HeLa cells that express $NUDT5^{E112Q}$, cultured in dFBS-supplemented medium upon 20 μM hypoxanthine treatment. Images were captured at 30-second intervals for 1 hour.

Movie 7: Imaging of GFP-FGAMS (green) in ΔNUDT5 HeLa cells that express $NUDT5^{R70A}$, cultured in dFBS-supplemented medium upon 20 μM hypoxanthine treatment. Images were captured at 30-second intervals for 1 hour.

References and Notes

50. F. Adikusuma, C. Pfitzner, P. Q. Thomas, Versatile single-step-assembly CRISPR/Cas9 vectors for dual gRNA expression. *PLoS One* **12**, e0187236 (2017).
51. E. Aznauryan *et al.*, Discovery and validation of human genomic safe harbor sites for gene and cell therapies. *Cell Rep Methods* **2**, 100154 (2022).
52. N. E. Sanjana, O. Shalem, F. Zhang, Improved vectors and genome-wide libraries for CRISPR screening. *Nat Methods* **11**, 783-784 (2014).
53. Z. Gu, R. Eils, M. Schlesner, Complex heatmaps reveal patterns and correlations in multidimensional genomic data. *Bioinformatics* **32**, 2847-2849 (2016).
54. Z. Pang *et al.*, MetaboAnalyst 5.0: narrowing the gap between raw spectra and functional insights. *Nucleic Acids Res* **49**, W388-W396 (2021).
55. B. Faubert *et al.*, Lactate Metabolism in Human Lung Tumors. *Cell* **171**, 358-371 e359 (2017).
56. A. Luengo *et al.*, Increased demand for NAD(+) relative to ATP drives aerobic glycolysis. *Mol Cell* **81**, 691-707 e696 (2021).
57. G. Wang *et al.*, PyMIC: A deep learning toolkit for annotation-efficient medical image segmentation. *Comput Methods Programs Biomed* **231**, 107398 (2023).
58. R. W. Yao, P. F. Luan, L. L. Chen, An optimized fixation method containing glyoxal and paraformaldehyde for imaging nuclear bodies. *RNA* **27**, 725-733 (2021).
59. F. Cai *et al.*, Comprehensive isotopomer analysis of glutamate and aspartate in small tissue samples. *Cell Metab* **35**, 1830-1843 e1835 (2023).
60. D. A. Lamprecht *et al.*, Targeting de novo purine biosynthesis for tuberculosis treatment. *Nature* **644**, 214-220 (2025).
